# Critical Role of Alternative M2 Skewing in miR-155 Deletion-Mediated Protection of Colitis

**DOI:** 10.3389/fimmu.2018.00904

**Published:** 2018-05-03

**Authors:** Jintao Li, Ji Zhang, Hongxia Guo, Shimin Yang, Weiping Fan, Nan Ye, Zhiqiang Tian, Tiantian Yu, Guoping Ai, Zigang Shen, Haiyang He, Ping Yan, Hui Lin, Xue Luo, Hongli Li, Yuzhang Wu

**Affiliations:** ^1^Institute of Tropical Medicine, Army Medical University, Chongqing, China; ^2^Department of Microbiology, College of Basic Medicine, Army Medical University, Chongqing, China; ^3^Institute of Immunology, PLA, Army Medical University, Chongqing, China; ^4^Department of Gastroenterology, Xinqiao Hospital, Army Medical University, Chongqing, China; ^5^Department of Microbiology and Immunology, Shanxi Medical University, Taiyuan, China; ^6^Department of Obstetrics and Gynecology, Southwest Hospital, Army Medical University, Chongqing, China; ^7^Department of Histology and Embryology, College of Basic Medicine, Army Medical University, Chongqing, China

**Keywords:** M2 macrophages, miR-155, colitis, C/EBPβ, SOCS1

## Abstract

Inflammatory bowel disease (IBD) is associated with dysregulation of both innate and adaptive immune response in the intestine. MicroRNA (miR)-155 is frequently expressed and functions in many immune cell types. Besides its function in adaptive immunity, miR-155 is a key regulator of the innate immune response in macrophages, dendritic cells, and even in epithelia cells. Although the roles of miR-155 within T and B lymphocytes in colitis have been reported, its function in innate immune cells has not been thoroughly examined. In this study, the dextran sulfate sodium (DSS)-induced colitis model was established in wild-type (WT) and miR-155^−/−^ mice. Our results showed that miR-155 deficiency in macrophages recapitulated the alleviated colitis feature of miR-155^−/−^ mice and appeared to skew toward the alterative M2 phenotype. Notably, the predominance of M2 in colon can result in dampened intestinal immune cell proliferation and inhibit CD4 T cell polarization toward Th1 and Th17. Moreover, C/EBPβ and SOCS1 were demonstrated as two key functional targets in this process. We also provided evidence for use of miR-155 inhibitor to treat colitis. Collectively, the findings highlight the central role of alternative M2 skewing for miR-155 function in colitis and reveal that macrophages might be a main target for therapeutics.

## Introduction

The intestine maintains an elaborate balance between immune responses and immune tolerance to microbiota, which depends upon diverse regulatory mechanisms. Inflammatory bowel diseases (IBDs), including ulcerative colitis (UC) and Crohn’s disease, are characterized by dysregulated intestinal immune response. The immune cells of the gut mucosa involved in IBD pathogenesis include the innate arm dendritic cells (DCs), macrophages, innate lymphoid cells (ILCs), and neutrophils, and the adaptive arm Foxp3^+^ regulatory T (Treg) cells, interferon-γ-producing type 1 helper T cells (Th1) and interleukin (IL)-17-producing helper T cells (Th17 cells) (1). Intestinal epithelial cells (IECs), which sense intestinal contents through surface receptors and secrete regulatory factors, are involved as well (2).

In the murine colitis model induced by dextran sulfate sodium (DSS), which mimics the hallmarks of IBD in human patients, DSS administration leads to increased intestinal permeability and subsequent invasion of intestinal microflora through epithelial cell damage. In the disease progression, mice treated with 3% DSS show significant influx of innate and adaptive immune cell populations into the colon, consecutively. Generally, DCs and macrophages accumulate rapidly in colon (from 5 to 12 days), while T and B cells infiltrate during the late phase (from 8 to 25 days) (3).

MicroRNAs (miRNAs) are a class of noncoding RNAs of approximately 21–23 nucleotides in length that regulate gene expression via binding to the 3′-untranslated region of target mRNA molecules at the posttranscriptional level and are critical for fine-tuning many biological processes. Studies of IBD patients and experimental colitis models have found related profiles of miRNAs in the colon. miR-155 is frequently expressed in multiple immune cell types and has pronounced effects on each cells’ functions and phenotypes. In adaptive immune responses, miR-155 regulates the differentiation and functions of Treg, Th17, CD8^+^ T cells (4), and T follicular helper cells (5) intrinsically. In innate immunity, the role of miR-155 is conflicting. In DCs, miR-155 has been characterized as a negative regulator of innate immune responses (6, 7), while in macrophages, miR-155 functions as a pro-inflammatory regulator by affecting NF-κB signaling (8) or by promoting M2 polarization (9).

In IBD, miR-155 is upregulated in inflamed lesions of patients with active UC (10, 11). However, the role of miR-155 in colitis is controversial. Researchers who produced miR-155^−/−^ mice observed that a proportion of miR-155^−/−^ mice develop spontaneous enteric inflammation (12), implying that miR-155^−/−^ mice are more susceptible to colitis. Yet, others have reported that miR-155^−/−^ mice have more resistance to DSS-induced colitis. It has also been shown that miR-155 may aggravate colitis (13) by directly inhibiting Th17 cell differentiation in CD4^+^ T cells (14) or by regulating IL-10-producing B cells (15).

Among the many innate immune cells in the intestine, macrophages are one of the most important types, differentiating from monocytes recruited from blood. Undifferentiated macrophages (M0) can polarize into pro-inflammatory M1 macrophages or anti-inflammatory alternative M2 macrophages due to intrinsic molecular regulators and differential extrinsic environment conditions. Macrophages are essential for maintaining intestinal homeostasis (16), acting *via* the production of retinoic acid and IL-10 to facilitate tolerance of the intestinal microbiota (17).

During colitis, inflammatory macrophages are recruited from blood monocytes in a CCR2-dependent manner, accumulating in the inflamed mucosa and producing pro-inflammatory mediators in the early stages of an immune response (18). If the inflammatory macrophage response is not controlled, the adaptive immune responses are evoked subsequently and inflammatory T cells, including Th1 cells and Th17 cells, are recruited to the pathogenic site. These cells can further lead to aggravated colitis. In some conditions, macrophages in the intestine could be forced to shift to the M2 phenotype by intrinsic factors and extrinsic factors. The induced M2 macrophages have shown therapeutic potential in chemical-induced colitis (19, 20), partially by producing IL-10 and arginase-1.

In this study, due to the importance of innate immune cells in colitis (21) and their ability to evoke adaptive immunity, we focused on investigating miR-155 function in innate immune cells and further exploring the precise mechanism underlying such. We found that miR-155 functions as a strong regulator of macrophage polarization in colitis and that its deficiency can lead to a shifting from M1 to M2. The M2 macrophage phenotype caused by miR-155 deficiency is able to further dampen intestinal inflammation by affecting adaptive effectors cells in colon lamina propria. Collectively, we highlight the role of M2 macrophages in miR-155-mediated orchestration of the intestinal immune response and intestinal inflammation.

## Materials and Methods

### Mice

WT C57BL/6 (B6), B6.Cg-Mirn155tm1.1Rsky/J (miR-155^−/−^), and CD45.1^+^ B6 mice were purchased from Jackson Laboratory and housed under specific pathogen-free conditions. Female mice of 8–10 weeks old were used for the experimental colitis modeling. All miRr-155 knockout mice used in our study were homozygous.

### Induction and Treatment of DSS Colitis in Mice

DSS-colitis was induced by 3% (w/v) DSS (Sigma-Aldrich) in drinking water for 5 days, followed by regular drinking water for 6 days. The clinical score of the disease activity index (DAI) (22) was used as the primary parameter to assess the severity of colitis. DAI was monitored every day in DSS-treated mice, consisting of loss of body weight (0, none; 1, 1–5%; 2, 5–10%; 3, 10–20%; 4, >20%), stool consistency (0, normal stool; 2, loose stool; 4, diarrhea), and hemoccult (0, normal; 2, hemoccult positive; 4, gross blood). In some experiments, liposome-mediated macrophage depletion was carried out as described previously (23). MC-21-mediated blood monocyte depletion was carried out as described previously (18). Broad-spectrum antibiotics were administered as in previous studies (24) to deplete commensal bacteria. IL-13 and IL-4 neutralizing antibodies as well as PGE2 inhibitor were used to block their respective functions (25). Briefly, liposome-mediated macrophage depletion was carried out with intraperitoneal (i.p.) injection of 200 µL clodronate-liposomes (Vrije Universiteit Amsterdam) at pre-DSS treatment days (-)4 and (-)1 and on treatment days 1, 3, and 5. MC-21 (CCR2 antibody)-mediated blood monocyte depletion was carried out with i.p. injection at indicated time points. Broad-spectrum antibiotics were administered as an antibiotic cocktail (4-week course, in drinking water) with vancomycin hydrochloride (0.5 g/L), neomycin sulfate (1.0 g/L), ampicillin (1.0 g/L), and metronidazole (1.0 g/L). For IL-4 and IL-13 neutralization, mice were i.p. injected with either 200 μg/mouse of anti-IL-4 neutralizing antibody (BioLegend) or 30 μg/mouse of anti-IL-13 neutralizing antibody (Peprotech) at treatment days 1, 3, and 5. PGE2 was blocked by daily i.p. injection of a PGE2 inhibitor (1 µg/g body weight in 200 µL phosphate buffered saline (PBS); Cayman Chemical) at treatment days 1, 3, and 5.

### Isolation of Lamina Propria Mononuclear Cells (LPMCs) and IECs

Briefly, the colon was removed from the sacrificed mice, cut into 0.5 cm pieces, and washed thoroughly with cold PBS to remove all debris and blood. IECs were obtained after incubating with 2 mM dithiothreitol (DTT) and 1 mM EDTA in PBS at 37°C for 2 × 20 min under gentle shaking. Then, the tissues were digested in 10 mL 2% fetal bovine serum (FBS)-RPMI-collagenase A (1 mg/mL; Roche) at 37°C for 30 min. Lamina propria cells were then collected and further purified *via* density gradient centrifugation with 40 and 70% Percoll–RPMI solution. LPMCs were collected from the interphase. The cell viability was determined with the CASY-TT cell counter and analyzer (Innovates-Roche). Viable single-cell suspensions were subjected to flow cytometric analysis or sorting.

### Generation of Bone Marrow Chimeric Mice

Bone marrow cells were harvested by flushing femurs and tibias from WT and miR-155^−/−^ mice that expressed either CD45.1 or CD45.2. Recipient mice were sublethally irradiated twice (8 Gy, 2 h apart), then bone marrow cells (2 million cells/mouse) were tail vein-injected into WT and miR-155^−/−^ mice. Four chimera groups were generated: WT → WT (WT cells expressing CD45.2 into WT mice expressing CD45.1), miR-155^−/−^ → WT (miR-155^−/−^ cells expressing CD45.2 into WT mice expressing CD45.1), WT → miR-155^−/−^ (WT cells expressing CD45.1 into miR-155^−/−^ mice expressing CD45.2), and miR-155^−/−^ → miR-155^−/−^ (miR-155^−/−^ cells expressing CD45.2 into miR-155^−/−^ mice expressing CD45.2). Post-transplantation, recipient mice underwent oral antibiotic treatment (weeks 1–2), engraftment recovery (weeks 3–6), and harvesting of peripheral blood leukocytes to assess degree of bone marrow reconstitution (week 7) by staining with anti-CD45.1 and anti-CD45.2 antibodies (BD Biosciences). The chimeric mice were considered to have been generated successfully once 90% of the hematopoietic cells were derived from donor bone marrow.

### Hematoxylin and Eosin (H&E) Staining and Immunohistochemistry

Colon tissue was fixed overnight (4% paraformaldehyde), embedded (paraffin), and sectioned (4 µm). Colon tissue sections were incubated with rat to mouse F4/80 antibody (Abcam), rabbit to mouse Arg1 antibody, and inducible nitric oxide synthase antibody (Abcam). Inflammation extent was scored on H&E-stained sections by investigators blinded to the experimental protocol. Immunofluorescence images were captured under fluorescence microscope (Leica) at identical exposure and intensity settings. All histological scorings and quantifications were performed in a blinded fashion.

### Histological Pathology Scoring

H&E-stained intestinal sections were scored by an expert blinded to the genotypes using a system based on parameters of tissue damage (degrees of crypt hyperplasia, epithelial injury/erosion, and edema; 0: absent, 1: slight, 2: moderate, and 3: severe), inflammation [numbers of mononuclear cells, polymorphonuclear cells, and lymphocytic cells (0: absent, 1: slight, 2: moderate, and 3: severe; multiplied by a factor accounting for the extent of tissue affected) and location of the inflammatory infiltrate (0: absent, 1: mucosal, 2: submucosal, 3: transmural extending into muscularis and serosa, and 4: diffuse)]. The tissue damage or inflammation score sum was multiplied by a factor according to the fraction of tissue affected (1: <10%, 2: 10–25%, 3: 25–50%, and 4: >50%).

### Bone Marrow-Derived Macrophage (BMDM) and Bone Marrow-Derived Dendritic Cells (BMDCs) Generation and Adoptive Transfer

Bone marrow cells isolated from femurs and tibias of WT and miR-155^−/−^ mice were depleted of erythrocytes and seeded in petri dishes (2 × 10^5^/mL). For BMDCs, the cells were differentiated in the presence of recombinant mouse GM-CSF (20 ng/mL; Peprotech) at 37°C in a CO_2_ incubator. The culture media were changed twice (on days 3 and 6), and on day 7 the non-adherent cells (DCs) were collected for *in vitro* experimentation (90-95% CD11c^+^ cells). For BMDMs, the cells were cultured in complete medium supplemented with 30% L929 cell culture supernatant (conditioned media). After 7 days, the adherent cells were harvested, washed, resuspended in complete medium, and used for transfection (>95% F4/80^+^). The BMDMs were detached using 10 mmol/L EDTA in PBS, washed 3 times with saline, and intravenously injected at 5 million cells/mouse into WT or miR-155^−/−^ mice at indicated time points. For the DC adoptive transfer experiment, BMDCs were treated with 100 ng/mL lipopolysaccharide for 3 h and then washed with PBS twice to remove the lipopolysaccharide. Stimulated BMDCs (at 5 million cells/mouse) were intravenously injected into DSS-treated mice at indicated time points.

### *In Vitro* Induction of BMDMs or Monocytes

WT and miR-155^−/−^ mature adherent BMDMs or sorted Ly6C^hi^ monocytes were primed with fresh medium and then treated with 10 µg/mL cecal bacterial antigen (CBA) and 20 ng/mL interferon (IFN)-γ or 15 ng/mL recombinant IL-4 (Peprotech) for 10 h. CBA was prepared using C57Bl6 mice as previously described, with minor modifications (26).

### miR-155 Knockdown *In Vivo* by Antagomir

Antagomirs are single-stranded oligonucleotides used to silence endogenous miRNAs. The terminal nucleotides at both ends of antagomirs are modified by an O-methyl moiety at the 2′-ribose position. Another modification is cholesteryl functionality at the 5′ end of the RNA at the sense strand. Antagomir-155 and scrambled controls were purchased from Ruibo Biotech, China and administered [5 mmol/kg/mouse by intravenous (i.v.) injection] to DSS-induced colitis mice on days 5 and 10 after challenge with DSS.

### Enzyme-Linked Immunosorbent Assay (ELISA)

Segments of colon tissue (1 cm each) were washed in cold Hank’s balanced salt solution supplemented with penicillin and streptomycin. These colon tissue segments were then cut into small pieces and cultured in 24-well flat-bottom culture plates (Falcon) in serum-free 1640 RPMI medium (Invitrogen). High concentration of penicillin and streptomycin was used to prevent bacteria growth. After incubation at 37°C for 24 h, the supernatant was collected and centrifuged at 13,000 *g* for 10 min at 4°C and stored at −80°C until subsequent ELISA analysis of cytokines. The ELISA kits for TNF-α, IL-17A, IL-1β, IL-12, and IFN-γ were obtained from BioLegend, and the PGE2, IL-10 ELISA kits were obtained from R&D Systems. The concentration of each of these cytokines was determined according to the manufacturer’s instruction and was normalized by total colon tissue weight of whole-colon culture.

### Cytokine PCR Array and miR-155 Target Array

Total RNA from the colons of WT and miR-155^−/−^ mice (*n* = 3) was prepared using the TRIzol reagent (Invitrogen). The samples’ quality was detected by BioRad NanoDrop 2000, and the A260/A280 ratio of 1.8~2.1 and the A260/A230 ratio were used in the real-time PCR experiments. The production of cytokines in colon was examined by the Innate & Adaptive Immune Responses PCR Array kit (Qiagen, Cat. No. PAHS-052Z). The expressions of 84 target genes of miR-155 were determined by the Mouse miR-155 Targets RT^2^ Profiler PCR Array (Qiagen). All arrays were analyzed in a Bio-Rad CFX96 according to the manufacturer’s instruction.

### Q-PCR Assay of miR-155 and M1/M2-Related Genes

For Q-PCR assay of miR-155, miR-155 primers and its control U6 primers and related kits (miRNA isolation kit and miRNA Q-PCR kit) were purchased from Applied Biosystems and used according to the manufacturer’s instruction. The ΔΔCT method was used to normalize data. For Q-PCR assay of genes, primer sequences used in this study are listed in Table S3 in Supplementary Material. Total RNA was extracted from colon tissues or cells as described above, and cDNA was synthesized with Power SYBR Green PCR Master Mix (Toyobo Co.).

### Flow Cytometry Analysis

For flow cytometry analysis or sorting, isolated splenocytes, LPMCs, or peripheral blood monocytes were fixed directly and surface-stained for CD45.1, CD45.2, CD3, CD4, CD8, CD115, Ly6G, Ly6C, CD11b, and CD11c. After permeabilization with 0.1% saponin (Sigma-Aldrich) in staining buffer, the cells were stained with specific fluorochrome-conjugated monoclonal antibodies (mAbs) against IFN-γ and IL-17A, etc. For cell sorting, CD4^+^ T cells and colon lamina propria macrophages (CD11b^+^CD11c^−/low^) were sorted from the isolated LPMCs, and Ly6C^hi^ monocytes (CD45^+^CD115^+^Ly6G^-^Ly6C^hi^) were sorted from blood. All antibodies were from eBioscience, unless otherwise indicated. Flow cytometry analysis was carried out using BD FACSCantoII and sorting with a BD FACSAria. Data were analyzed by FlowJo software (v9.2; TreeStar).

### Human Tissue Samples

This study was approved by the Ethics Committee of Xinqiao Hospital of Third Military Medical University. Colorectal tissue samples obtained, with written informed consent, included sigmoid colon of 22 patients exhibiting no apparent intestinal pathology and normal mucosa (>10 cm colitis margin) of 20 patients undergoing anterior resection, more details about the patients are listed in Table S1 in Supplementary Material.

### Statistical Analysis

Quantitative data are presented as mean ± SD of at least three experiments. Survival curves were plotted according to the Kaplan–Meier method and compared using the log-rank test. Two-tailed Student’s *t*-test or ANOVA with Bonferroni’s posttest correction for multiple comparisons were used for testing significance. All statistical analyses were performed with Prism 6.0 for Windows (GraphPad).

## Results

### Reduced Innate and Adaptive Immunity in Colon of miR-155^−/−^ Mice in Acute Colitis

**Figure 1:**
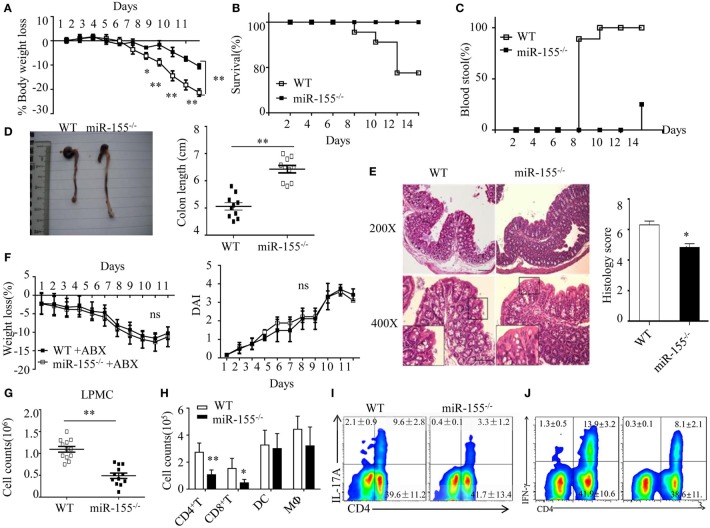
Attenuated dextran sulfate sodium (DSS)-induced colitis in miR-155^−/−^ mice is dependent on commensal bacteria. WT or miR-155^−/−^ mice were given 3% DSS in drinking water for 5 days, followed by regular drinking water for 6 days. **(A–E)** Weight change **(A)**, Kaplan–Meier plot of survival rate **(B)**, stool blood **(C)**, representative gross colon appearance [**(D)** left] and colon length [**(D)** right], and representative H&E-stained colon cross-sections [**(E)** left, original magnification, 200× or 400×] and semi-quantitative histopathology score [**(E)** right]. **(F)** WT and miR-155^−/−^ mice were treated with broad-spectrum antibiotics cocktail (ABX) for 4 weeks and then given 3% DSS, the body weight change (left) and DAI (right) were monitored daily. Ns vs WT control **(G,H)**. The LPMCs were isolated from colon tissues of DSS-treated WT (*n* = 12) and miR-155^−/−^ (*n* = 15) mice, then the total number of LPMCs (CD45^+^) **(G)**, T cells (CD4^+^ and CD8^+^), DCs (CD11c^+^CD11b^−^) and macrophages (CD11b^+^CD11c^−/low^) **(H)** were counted by flow cytometry. **(I,J)** Representative FACS showing CD4^+^IL-17^+^ cells **(I)** and CD4^+^IFN-γ^+^ cells **(J)** in isolated LPMCs of DSS-treated WT (*n* = 12) and miR-155^−/−^ (*n* = 15) mice. *P < 0.05, **P < 0.01 vs WT control [Student’s *t*-test in **(A,D,E,G,H)** and Kaplan–Meier analysis in **(B,C)**]. ns vs WT control. Data are representative of three independent experiments (mean and SD in A-D); *n* = 12–15 mice per group in **(A–F)** and *n* = 5–6 mice per group in **(G)**. ns, not significant. WT, wild-type.

miR-155 expression was significantly increased in mouse colon tissues, IECs and LPMCs (Figure S1A–C in Supplementary Material) during DSS-induced colitis, as well as in human UC colon tissue (Figure S1D in Supplementary Material). The miR-155^−/−^ mice showed significant protection from DSS-induced colitis (vs WT mice), according to body weight loss, survival, rectal bleeding, colon length and histopathology (Figures 1A–E, respectively). The similar results obtained from the 2,4,6-trinitrobenzene sulfonic acid colitis model excluded a model-specific effect of miR-155 in acute colitis (Figure S2 in Supplementary Material).

We next examined whether the phenotype in miR-155^−/−^ mice was dependent on commensal bacteria. We found that pre-depletion of microbiota by broad-spectrum antibiotics abolished the observed phenotype of miR-155^−/−^ mice (becoming comparable to that of WT), as assessed by body weight loss and DAI (Figure 1F). Furthermore, we investigated the possibility that miR-155 affected DSS-induced colitis by altering intestinal microbial ecology (27). Total bacteria numbers and three representative groups (*E. rectale–C. coccoides, Bacteroides* spp., and *Enterobacteriaceae*)^25^ in stool of WT and miR-155^−/−^ mice were similar between the mice (Figure S3 in Supplementary Material). Thus, the miR-155-mediated colitis phenotype is driven by microbiota but not *via* alteration of its composition.

Since IEC function contributes to the pathogenesis of DSS-induced colitis (28), we first compared the non-immune character of IECs in WT and miR-155^−/−^ mice. However, neither permeability nor apoptosis or proliferation (Figure S4A–C in Supplementary Material) was observed to differ significantly, suggesting that miR-155 may not contribute to DSS-induced colitis through non-immune mechanisms. We, thus, subsequently examined the production of cytokines in colon (explants) using PCR array and ELISA, respectively, to investigate the potential immune factors in this miR-155 mediated process. The colonic explants from miR-155^−/−^ mice expressed significantly lower amounts of inflammatory cytokines and increased amounts of anti-inflammatory cytokines (Figures S5A,B in Supplementary Material). miR-155^−/−^ mice had lower number of LPMCs (CD45^+^) (Figure 1G), in which CD4^+^ and CD8^+^ T cells were reduced and macrophages (CD11b^+^CD11c^−/low^) and DCs (CD11c^+^CD11b^−^) were not significantly affected (Figure 1H). Moreover, DSS-treated miR-155^−/−^ mice showed a lower frequency of Th17 and Th1 cells in the lamina propria (vs DSS-treated WT; Figures 1I,J). Collectively, these observations suggest that in DSS-induced colitis, miR-155^−/−^ in mice results in reductions in both innate immunity and adaptive immunity in the intestine.

### Lack of miR-155 in Hematopoietic Cells, Rather Than in Non-Hematopoietic Cells, Recapitulates the Phenotype of Global miR-155 Deletion

**Figure 2:**
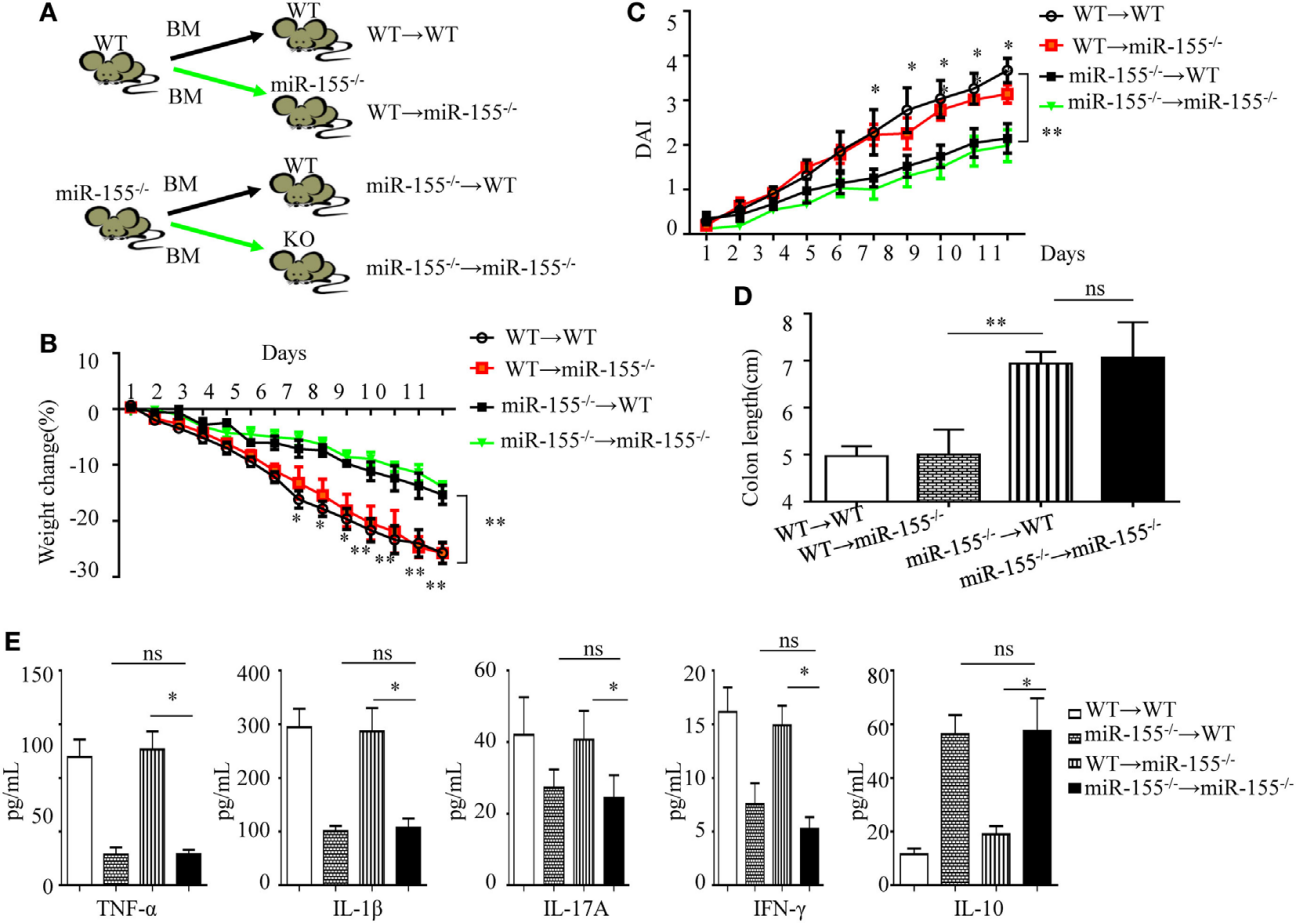
miR-155 deficiency ameliorates colitis by affecting inflammatory signaling in mucosal immune cells. The bone marrow chimeras were reconstructed and 7–8 weeks later treated with 3% dextran sulfate sodium as in Figure 1A. **(A)** Schematic of bone marrow chimera reconstructions. **(B–D)** Body weight **(B)** and DAI **(C)** were monitored daily, and colon length of each bone marrow chimeras was examined at day 11**(D)**. **(E)** The cytokine concentrations in the supernatants of 24 h cultured colon explants were assessed by ELISA and were normalized by total colon tissue weight in whole-colon culture. *P < 0.05, **P < 0.01, WT → miR-155^−/−^ mice vs miR-155^−/−^ → miR-155^−/−^ mice. ns, not significant, miR-155^−/−^ → WT mice vs miR-155^−/−^ → miR-155^−/−^. Student’s *t*-test in **(A–D)**; *n* = 11–14 per group. Data are representative of two independent experiments (mean and SD). BM, bone marrow; WT, wild-type.

Following DSS challenge, colonic mucosal immunity can be induced through pattern-recognition receptors in both IECs (derived from non-hematopoietic cells) and/or mucosal immune cells (derived from hematopoietic cells) (29). To determine their role in miR-155^−/−^ mice colitis, we generated bone marrow chimeras to assess the target cell type of miR-155 function. Irradiated C57BL/6 mice were transplanted with bone marrow from WT (WT → WT) or miR-155^−/−^ (miR-155^−/−^ → WT), and irradiated miR-155^−/−^ mice were transplanted with that from WT (WT → miR-155^−/−^) or miR-155^−/−^ mice (miR-155^−/−^ → miR-155^−/−^), respectively (Figure 2A). At 6 weeks posttransplantation, >90% of blood leukocytes were of donor origin, confirming successful reconstitution (Figure S6 in Supplementary Material). The miR-155^−/−^→ WT mice with hematopoietic miR-155 deficiency showed attenuated severity of DSS-induced colitis similar to miR-155^−/−^ → miR-155^−/−^ mice, based on body weight loss, DAI, colon length and pro-inflammatory cytokine levels in the colon (Figures 2B–E, respectively, and Figure S7 in Supplementary Material). In contrast, the WT → miR-155^−/−^ mice, having miR-155 deficiency in non-hematopoietic cells, developed more severe colitis than the miR-155^−/−^→ miR-155^−/−^ mice based on the same parameters. Thus, these results indicate that miR-155 deficient in mucosal immune cells that were derived from hematopoietic cells rather than in non-hematopoietic cells, accounted for the observed reduction in colonic inflammation in the miR-155^−/−^ mice.

### Effects of miR-155 During Acute Colitis Are Predominately Attributed to Its Function in Macrophages

**Figure 3:**
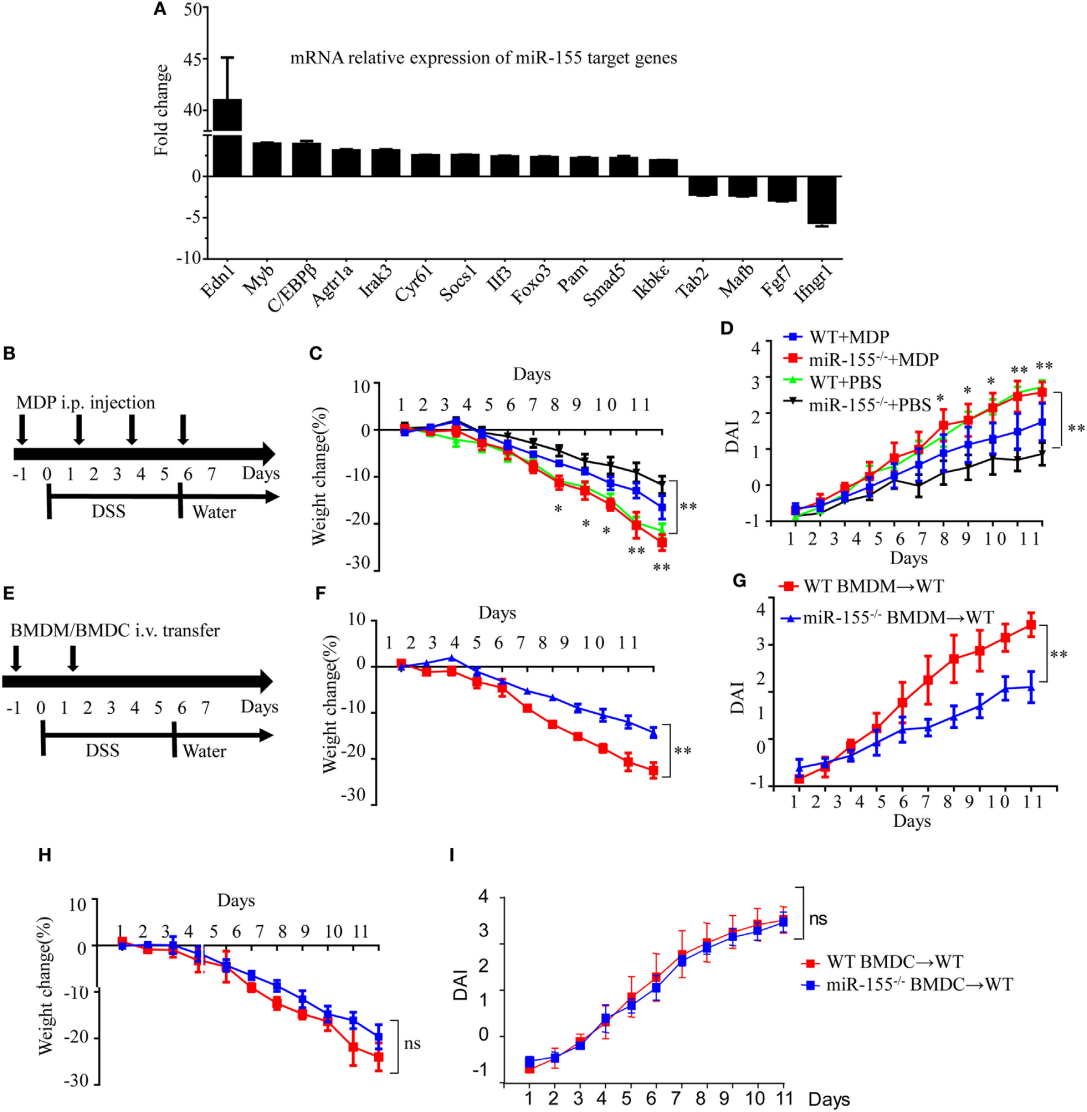
Macrophages play a critical role in miR-155 deficiency-mediated suppression of dextran sulfate sodium (DSS)-induced colitis. WT (*n* = 3) or miR-155^−/−^ (*n* = 3) mice were given 3% DSS in drinking water for 5 days, followed by regular drinking water for 6 days. **(A)** Total RNA was extracted from colon tissues and analyzed by the mouse miR-155 Targets RT^2^ Profiler PCR Array (Qiagen), and the most differentially expressed target genes were ranked. **(B–D)** WT (*n* = 12) or miR-155^−/−^ (*n* = 13) mice were administrated intravenously with clodronate-liposomes (MDP) or control PBS-liposomes (PBS) as schematic protocol indicated during DSS colitis induction **(B)**. Body weight **(C)** and DAI **(D)** were monitored daily. *P < 0.05, **P < 0.01 (WT + MDP) vs (miR-155^−/−^ + MDP) (Student’s *t*-test). **(E)** WT (*n* = 12) mice were adoptive transferred intravenously with 5 million WT or miR-155^−/−^ BMDMs (BMDCs) as the schematic protocol indicated during DSS colitis induction. Body weight changes **(F)** and DAI **(G)** of WT mice with transferred WT or miR-155^−/−^ BMDMs were monitored throughout the study. **P < 0.01, (miR-155^−/−^ BMDM → WT) vs (WT BMDM → WT) (Student’s *t*-test). **(H,I)** Body weight changes **(H)** and DAI **(I)** of WT mice with transferred WT or miR-155^−/−^ BMDCs were monitored throughout the study. ns (miR-155^−/−^ BMDCs → WT) vs (WT BMDCs → WT) (Student’s *t*-test). Data are representative of two independent experiments (mean and SD). WT, wild-type. MDPs clodronate-liposomes. BMDMs, bone marrow-derived macrophages. BMDCs, bone marrow-derived dendritic cells.

Assuming that miR-155 deficiency increases the expression of its direct targets, we compared the expression of 84 miR-155 target genes in colon tissues after establishment of DSS colitis. C/EBPβ, a direct target of miR-155 in macrophages and a key regulator of M2 polarization (30), was among the most increased genes in miR-155^−/−^ mice after DSS exposure (vs WT; Figure 3A), implicating macrophages and their M2 polarization in miR-155 deletion-mediated protection. The body weight loss and DAI features of macrophage-depleted miR-155^−/−^ mice (by clodronate-liposome treatment, Figure S8 in Supplementary Material) (31) in colitis were similar to those in WT mice (Figures 3B–D), demonstrating that macrophage depletion abrogates the effects of miR-155 in acute colitis.

To further determine whether macrophages in the miR-155 deficient mice were sufficient to achieve protection similar to that seen in miR-155 global knockout in DSS-induced colitis, miR-155^−/−^, or WT BMDMs were adoptively transferred into WT recipients, and disease severity was examined. Indeed, recipients of miR-155^−/−^ BMDMs showed significantly reduced disease activity compared to mice that received WT BMDMs (Figures 3E–G). However, mice that received adoptively transferred miR-155^−/−^ BMDCs or WT BMDCs exhibited a similar degree of decreased disease activity following DSS exposure (Figures 3H–I). Thus, miR-155 activity in macrophages is primarily responsible for its effect in reducing severity of DSS-induced acute colitis.

### miR-155^−/−^ Intestinal Macrophages Are Prone to M2 Phenotype in DSS-Induced Colitis

**Figure 4:**
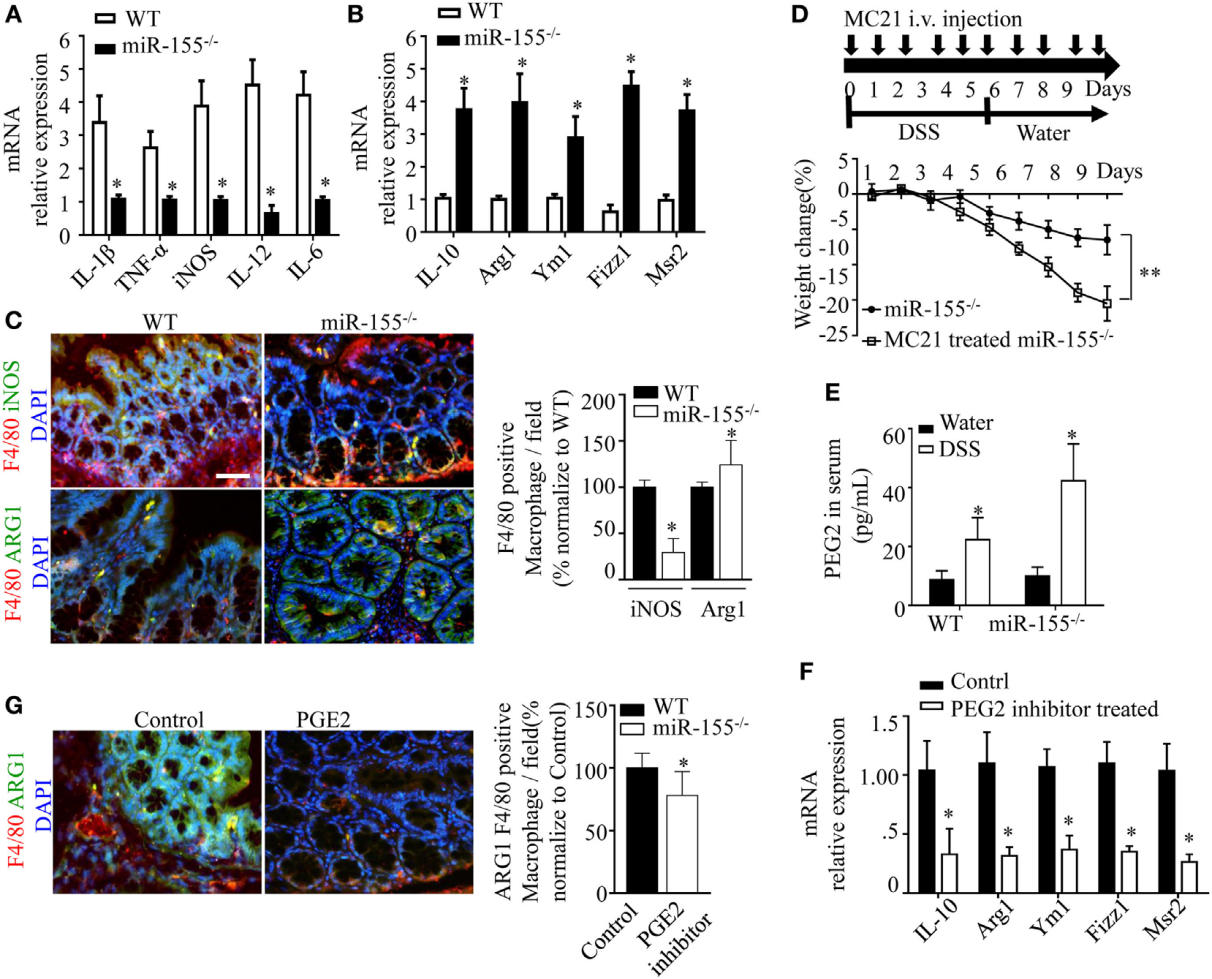
miR-155 ablation suppresses M1 polarization and reciprocally promotes M2 phenotype in dextran sulfate sodium (DSS)-induced colitis. **(A–C)** Total RNA was isolated from colon tissues of WT (*n* = 5) or miR-155^−/−^ (*n* = 5) mice that were given 3% DSS in drinking water for 5 days, followed by regular drinking water for 6 days. The relative expression of mRNA associated with M1-polarized macrophages **(A)** and M2-polarized macrophages **(B)** was measured by Q-PCR. **(C)** Representative immunofluorescence images (left) and the quantification (right) of M1 and M2 in colon tissues co-stained with macrophage F4/80 (green), iNOS/Arg1 (red), and DAPI (nuclei, blue). **(D)** In miR-155^−/−^ mice (*n* = 8), depletion of Ly6C^hi^ monocytes in the blood was achieved using MC21 antibody during DSS colitis induction as the schematic protocol indicated (up) resulted in aggravated DSS symptoms (down). **(E)** Serum levels of PEG2 in WT (*n* = 5) and miR-155^−/−^ (*n* = 5) mice at day 2 of DSS colitis were determined by ELISA. **(F–G)** miR-155^−/−^ mice (*n* = 5/group) were i.p. injected with PGE2 inhibitor at DSS treatment days 1, 3, and 5. The relative expressions of M2 genes were measured by Q-PCR **(F)**, and the number of M2(F4/80^+^ Arg1^+^) was determined by immunofluorescence **(G)**. *P < 0.05 vs WT control [Student’s *t*-test in **(A–G)**]. Data are representative of two or three independent experiments (mean and SD). ns, not significant; WT, wild-type; CBA, cecal bacterial antigen; Arg1, arginase-1.

Prompted by the sharp upregulation of the M2 polarization-associated transcription factor, C/EBPβ, in colon of miR-155^−/−^ mice with acute colitis, we hypothesized that the alternative activated M2 phenotype might represent the predominant macrophages in miR-155^−/−^ colon. Indeed, M2-associated genes (Arg1, IL-10, Fizz1, and Mrc1) were increased and M1-associated genes (IL-1β, IL-6, IL-12, and TNF-α) were decreased in colons of DSS colitis miR-155^−/−^ mice (Figures 4A,B). The M2 macrophages predominance was confirmed by immunofluorescence (Figure 4C).

During colitis, pro-inflammatory M1 lamina propria macrophages are recruited from Ly6C^hi^ blood monocytes in a CCR2-dependent manner (18). To determine whether the anti-inflammatory M2 macrophages in miR-155^−/−^ mice were derived from this precursor cell type, the blood Ly6C^hi^ monocytes (CD45^+^CD115^+^Ly6G^-^Ly6C^hi^) (32) (Figure S9 in Supplementary Material) were sorted and differentiated into M1 (*via* 10 µg/mL CBA (26) and 20 ng/mL IFN-γ) and M2 (*via* 15 ng/mL IL-4) populations *in vitro*. In both conditions, the differentiated cells from miR-155^−/−^ monocytes showed higher M2-like phenotype (vs cells from WT; Figure S10 in Supplementary Material). Next, peripheral blood Ly6C^hi^ monocytes of miR-155^−/−^ mice were depleted *in vivo via* i.v. injection of anti-CCR2 MC21 depleting mAbs. We found the MC21-treated miR-155^−/−^ mice showed more severe DSS colitis (vs untreated; Figure 4D). Thus, the colonic M2 macrophages of miR-155^−/−^ mice might differentiate directly from circulating Ly6C^hi^ monocytes in DSS-induced colitis.

M2 polarization is dependent on Th2 cell cytokines. Daily measurement of DSS-induced temporal change in serum levels of IL-4 and IL-13 in miR-155^−/−^ mice showed that these cytokines were undetectable until M2 cells appeared in the colon (DSS challenge day 5; data not shown). Anti-IL-4 and/or anti-IL-13 neutralizing antibodies did not decrease expression of M2 genes (Figure S11 in Supplementary Material). We observed that prostaglandin E2 (PEG2; an M2 polarization inducer) (33, 34) was markedly elevated in serum of both WT and miR-155^−/−^ mice from DSS challenge day 2 (Figure 4E). A PEG2 inhibitor (administered *via* i.p. injection) significantly reduced the DSS-induced colonic expression of M2 genes (Figures 4F–G). Thus, in the miR-155^−/−^ mice, blood Ly6C^hi^ monocytes might be recruited to the colon and differentiated toward an M2 phenotype through PEG2 in DSS-induced colitis.

### miR-155^−/−^ Macrophages Inhibit Inflammatory Cells and Establish a Th1/Th17-Suppressive Environment

**Figure 5:**
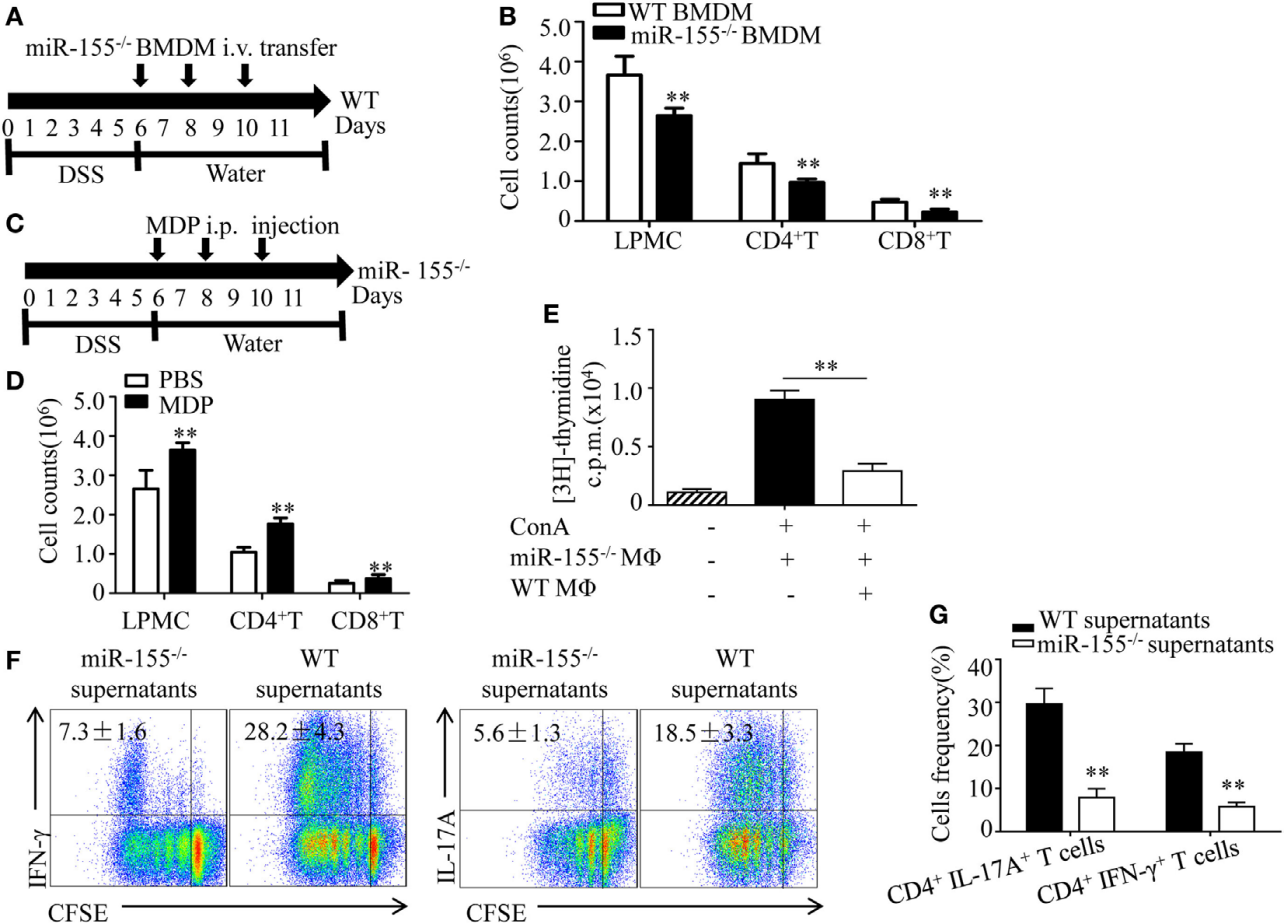
miR-155^−/−^ macrophages inhibit inflammatory cells and shape Th1/Th17 polarization **(A,B)** WT (*n* = 5) mice with dextran sulfate sodium (DSS) colitis were adoptive transferred with miR-155^−/−^ BMDMs or WT BMDMs (5 million/mouse) as schematic protocol indicated. The total number of LPMCs, CD4^+^ T cells, and CD8^+^ T cells of colon tissues at day 11 were counted. **(C,D)** miR-155^−/−^ mice (*n* = 5) with DSS colitis were i.p. injected with clodronate-liposomes (MDP) or control PBS-liposomes (PBS) as the schematic protocol indicated, and the total number of LPMCs, CD4^+^ T cells and CD8^+^ T cells of colon tissues at day 11 were counted. **(E)** OT-II mice splenocytes with incorporation of [^3^H]thymidine were cultured in the presence (+) or absence (−) of ConA, with (+) or without (−) miR-155 or WT BMDMs, then proliferation was assessed by [^3^H]thymidine incorporation. **(F)** CFSE-labeled naive OT-II T cells were cultured with OVA and miR-155^−/−^ or WT BMDMs culture supernatants for 4 days, and subjected to flow cytometry with gating of CD4^+^ T cells. **(G)** Flow cytometry of populations of CD4^+^IL-17^+^ T cells and CD4^+^IFN-γ^+^ T cells by OT-II T cells were primed as described in **(F)**, then allowed to “rest” and re-stimulated for 6 h with anti-CD3 and anti-CD28. Numbers in outlined areas indicate percent cells in the gate. *P < 0.05, **P < 0.01 vs WT control [Student’s *t*-test in **(B,D,E,G)**]. Data are representative of three independent experiments [mean and SD in **(A–D)**]; *n* = 12–15 mice per group in **(B,D)**. WT, wild-type.

We next examined how miR-155^−/−^ macrophages influence the pathogenesis of colitis. M2 macrophages suppress inflammatory cells during acute lung injury (35). To determine, in DSS-induced colitis, whether the observed decline in total numbers of LPMCs in miR-155^−/−^ mice (vs that in WT mice) were ascribable to M2 gene induction, we performed adoptive transfer of miR-155^−/−^ BMDMs into WT mice with DSS colitis and found that the total number of inflammatory cells was markedly reduced (vs WT BMDMs transfer; Figures 5A,B). In contrast, in the miR-155^−/−^ mice, when macrophages were depleted *via* clodronate-liposomes, the cell numbers increased (Figures 5C,D). Moreover, *in vitro* assays showed that miR-155^−/−^ macrophages exerted a great inhibitory effect on T cell proliferation (Figure 5E). Thus, these results suggest that miR-155^−/−^ macrophages can dampen the proliferation of immune cells that contribute to colitis.

Macrophages participate in intestinal immunity by producing mediators that direct T cell polarization (36, 37). To examine the effect of miR-155^−/−^ macrophages on T cell differentiation, OT-II CD4^+^ T cells labeled with carboxyfluorescein succinimidyl ester (commonly known as CFSE) were incubated *in vitro* and added to cell culture supernatants of BMDMs from WT and miR-155^−/−^ mice, respectively. We observed in naïve CD4^+^ T cells that adding supernatants from miR-155^−/−^ led to decreased frequency of Th17 and Th1 cells compared to that from WT (Figure 5F), indicating that miR-155^−/−^ BMDMs secrete factors to suppress CD4^+^ T cells polarized toward IFN-γ- producing Th1 cells and IL-17-producing Th17 cells. Re-stimulation with CD3- and CD28-specific antibodies enhanced their suppressive effect to this response (Figure 5G). These data suggest that miR-155^−/−^ macrophages can establish a Th1/Th17-suppressive environment.

### C/EBPβ and SOCS-1 Are Functional Targets in Intestinal Macrophage Polarization

**Figure 6:**
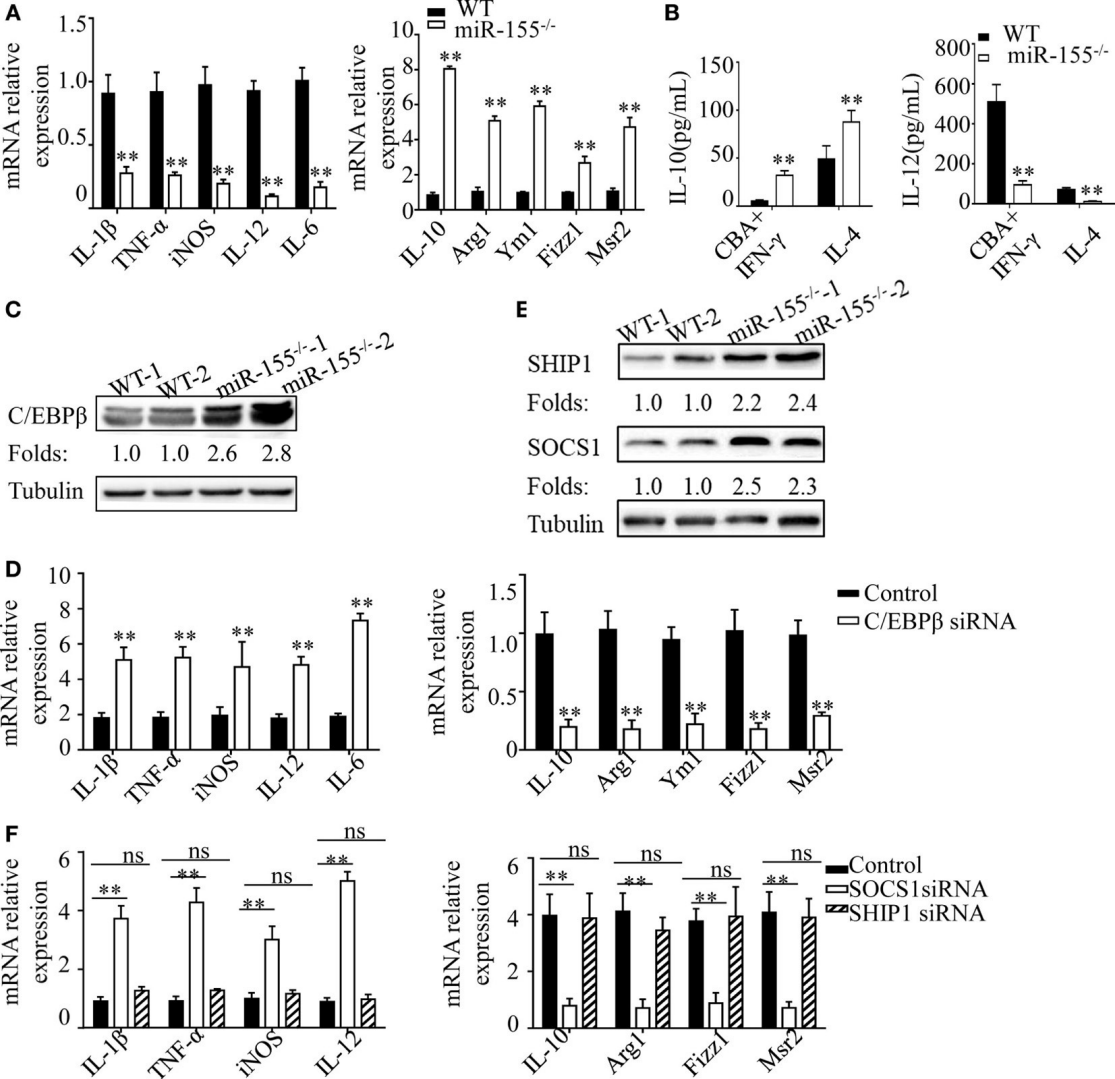
C/EBPβ and SOCS1 are key functional targets in intestinal M2 polarization. **(A)** BMDMs isolated from WT and miR-155^−/−^ mice were treated with CBA (10 µg/mL) and IFN-γ (20 ng/mL), and the relative expression of M1genes and M2 genes were determined by Q-PCR. **(B)** The absolute amounts of secreted cytokines IL-10 and IL-12 (as representative of M2 and M1 gene products, respectively) in the supernatants of WT or miR-155^−/−^ BMDMs that had been treated with M1 condition (CBA + IFN-γ) and M2 condition (IL-4) were measured by ELISA. **(C)** The protein expression level of C/EBPβ in macrophages (CD11b^+^CD11c^−/low^) isolated from LPMCs of dextran sulfate sodium colitis mice were determined by western blotting. **(D)** miR-155^−/−^ BMDMs were transferred with C/EBPβ siRNA or control and then stimulated with CBA (10 µg/mL) and IFN-γ (20 ng/mL), and the relative expression of M1genes and M2 genes were determined by Q-PCR. **(E)** The protein expression level of SOCS1 and SHIP1 in macrophages, as described in **(C)**, was determined by western blotting. **(F)** miR-155^−/−^ BMDMs were transferred with SOCS1 and SHIP1 siRNA and treated as described in **(D)**, and the relative expressions of M1genes and M2 genes were determined by Q-PCR. *P < 0.05, **P < 0.01 vs WT control or siRNA control [Student’s *t*-test in **(A,B,D)**]. *P < 0.05, ns > 0.05 vs. siRNA control (ANOVA with Bonferroni’s posttest correction for multiple comparisons in **(F)**. Data are representative of three independent experiments (mean and SD). Ns, not significant. BMDMs, bone marrow-derived macrophage. WT, wild-type. CBA, cecal bacterial antigen.

In DSS-induced colitis, intestinal commensal bacteria generated a M1-like condition for macrophage polarization. To explore the molecular mechanism through which miR-155^−/−^ promotes M2 polarization in this condition, we isolated BMDMs from WT and miR-155^−/−^ mice and treated with CBA (10 μg/mL)/IFN-γ (20 ng/mL) in an *in vitro* assay, to mimic intestinal bacteria stimulation. We found that miR-155^−/−^ BMDMs showed reduced expression of M1 genes and strongly increased expression of M2 genes (vs WT; Figure 6A). Moreover, absolute quantification of the amount of M2 and M1 gene products (represented by the secreted cytokines IL-10 and IL-12, respectively) by using ELISA showed that, even under M1-like polarization condition, BMDMs from miR-155^−/−^ mice can produce higher M2-related factors but with lower M1-related factors (vs WT; Figure 6B).

To determine the mechanism underlying the proclivity of miR-155^−/−^ macrophages to M2 polarization in this context, we first examined the protein levels of C/EBPβ, which is a target of miR-155 in M2 polarization (38). Colon lamina propria macrophages (CD11b^+^CD11c^−/low^) sorted from DSS challenged miR-155^−/−^ mice showed significantly upregulated C/EBPβ (vs WT; Figure 6C). We next examined whether small interfering (si)RNA-mediated knockdown of C/EBPβ expression could rescue the phenotype of miR-155^−/−^ macrophages under M1-like condition (CBA/IFN-γ treatment). C/EBPβ knockdown (Figure S12A in Supplementary Material) in miR-155^−/−^ BMDMs led to substantially reduced M2 gene expression and modestly increased M1 gene expression (Figure 6D). Thus, C/EBPβ is a key target of miR-155 in macrophage polarization, but is only partially responsible for miR-155 effects.

We further identified more targets of miR-155 involved in macrophage polarization. As miR-155 is a well-studied miRNA with a plethora of targets identified, we used a candidate approach to identify its target genes in macrophage polarization, with the following criteria: (1) prediction or experimental validation as targets of miR-155; and function as (2) negative regulator of M2 and positive regulator of M1. Two genes (SOCS-1and SHIP-1) met the criteria, and both were up-regulated in the miR-155^−/−^ BMDMs (Figure 6E). However, only SOCS-1 knockdown (Figures S12B,C in Supplementary Material) strongly restored M1 genes and slightly decreased M2 genes (Figure 6F). Thus, SOCS-1 is another miR-155 target involved in macrophage polarization, and it might primarily serve to mediate inaction of M1 genes.

### Anti-miR-155 Treatment Ameliorates DSS-Induced Acute Colitis *In Vivo*

**Figure 7:**
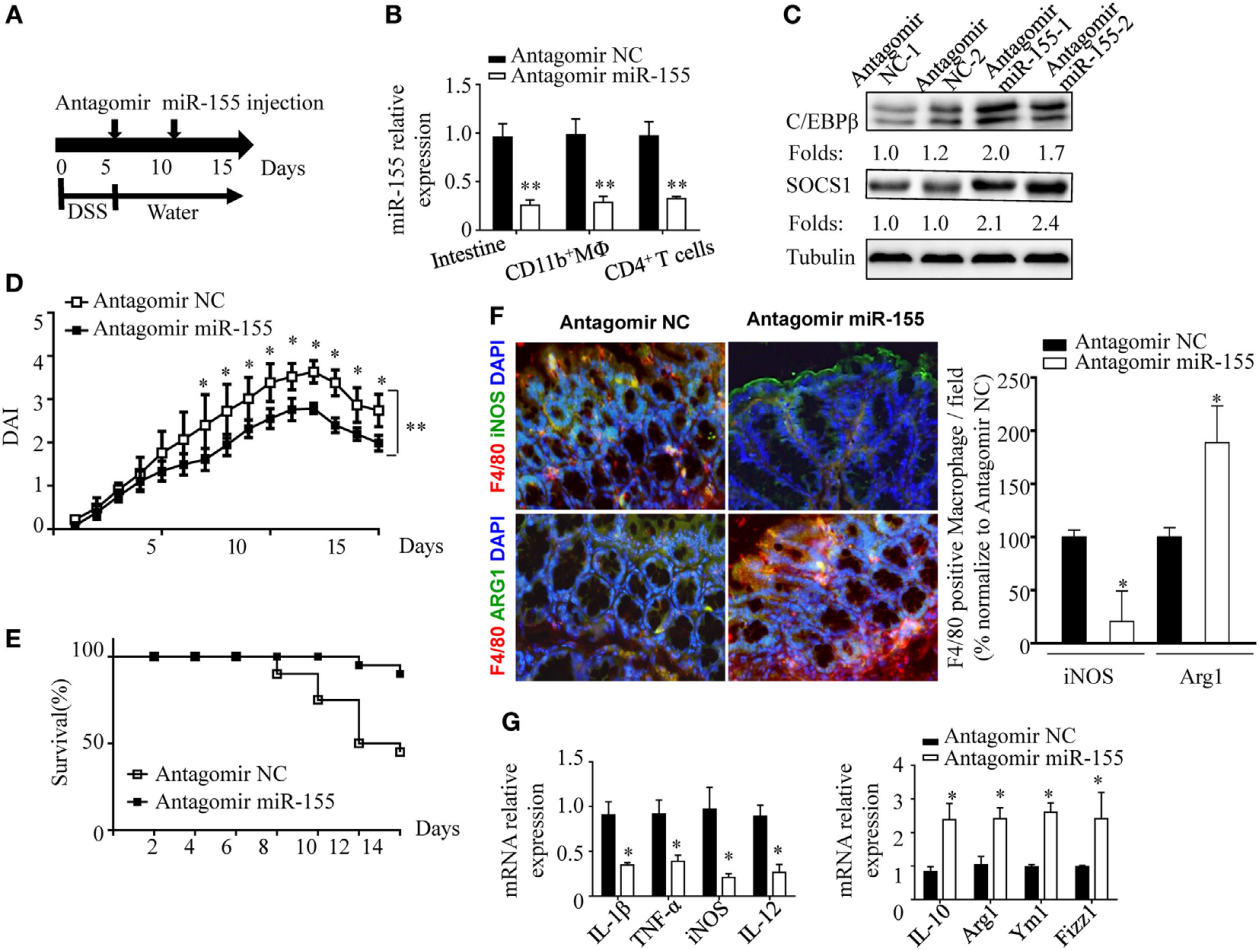
miR-155 antagomir treatment ameliorates dextran sulfate sodium (DSS) colitis pathogenesis and the proposed mechanism. **(A–C)** WT mice with DSS colitis were administered i.v. antagomir-155 (5 mmol/kg per mouse) or scrambled controls as the schematic protocol indicated, and miR-155 expression level and its functional targets C/EBPβ and SOCS1 were determined by Q-PCR and western blotting respectively. **(D,E)** The DAI **(E)** and survival rate **(E)** of WT mice with DSS colitis that were treated with antagomir-155 or of NC mice were monitored daily till day 15 after DSS challenge. **(F)** The representative immunofluorescence images (left) and the quantification (right) of M1 and M2 in colon tissues co-stained with macrophage F4/80 (green), iNOS/Arg1 (red), and DAPI (nuclei, blue). **(G)** The relative expression of M1 genes and M2 genes in colon of antagomir-155 treated mice at day 15. *P < 0.05, vs antagomir-treated NC (Student’s *t*-test in **(B,D,G)**]; *n* = 4–5 per group. Data are representative of two independent experiments (mean and SD). NC, negative control; WT, wild-type.

**Figure 8:**
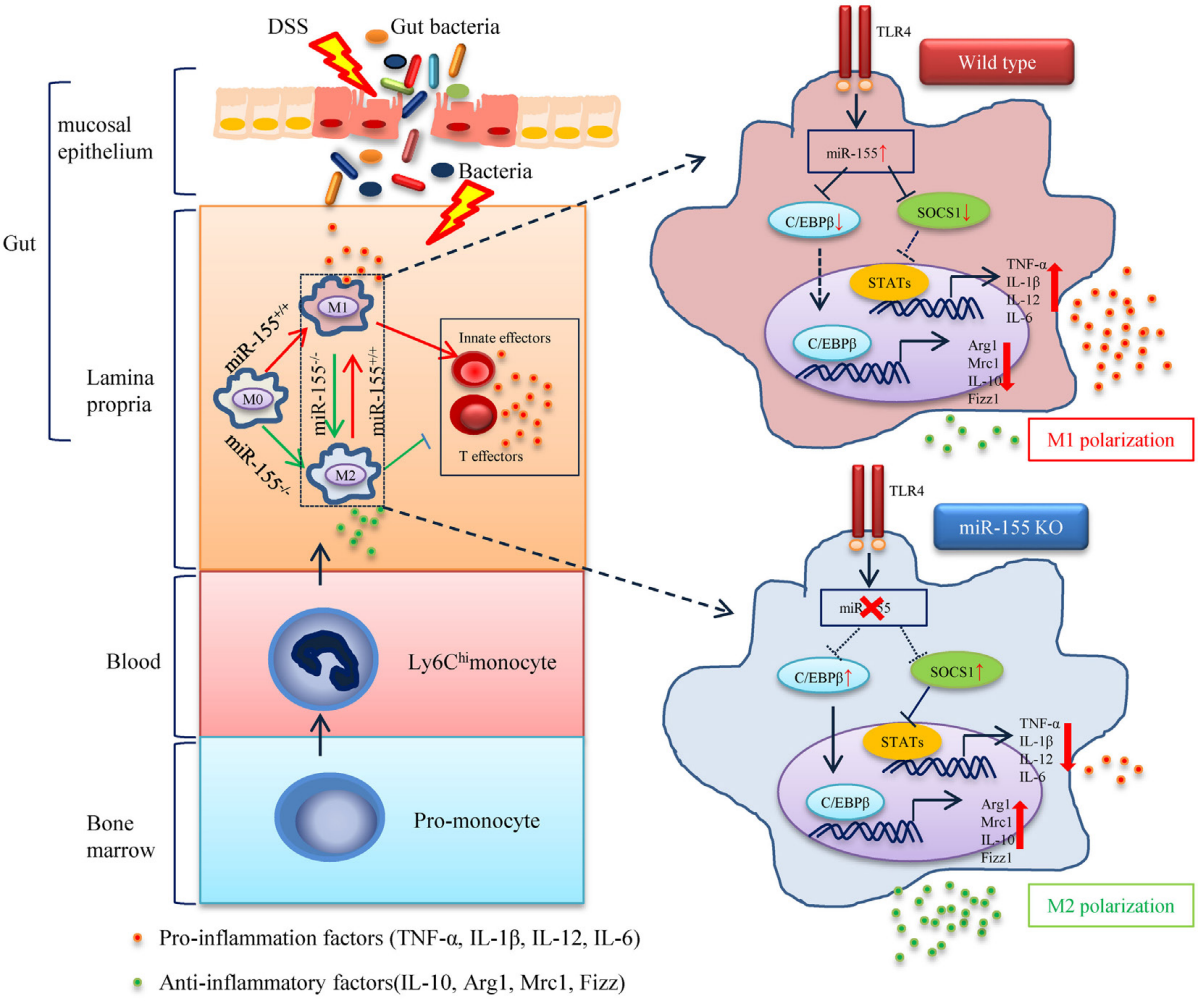
The proposed mechanism of miR-155 in the pathogenesis of dextran sulfate sodium (DSS)-induced colitis. In steady state, resident macrophages maintain tolerance toward the intestinal microbiota. Upon DSS exposure, gut bacteria break through the intestinal mucosal barrier and inflammatory macrophages are recruited from blood monocytes and accumulate in the inflamed mucosa, where they produce pro-inflammatory mediators. While in the absence of miR-155, two target genes C/EBPβ and SOCS1 in Ly6C^hi^ monocytes are unregulated and functional. This leads to the undifferentiated M0 macrophages shifting to the M2 phenotype in a PGE2-dependent manner, with a reduced M1 gene program expression and an enhanced M2 gene program expression. The miR-155 deficiency forced M2-like dominant macrophages in lamina propria to shape the colon environment and condition the proliferation and polarization of T cells, which facilitated the maintenance of intestinal homeostasis and resolved the pathogenesis of DSS-induced colitis.

The phenotypic observations in miR-155^−/−^ mice indicated the prophylaxis potential of anti-miR-155 in acute colitis; thus, we further examined the role of anti-miR-155 in the therapeutic context by using antagomirs (39) (Figure 7A). At 48 h after the antagomir-155 injection, miR-155 expression was efficiently silenced in intestinal tissues and colon immune cells (Figure 7B); meanwhile, C/EBPβ and SOCS1 protein expression was increased in the colon (Figure 7C). The antagomir-155 treatment substantially ameliorated clinical symptoms of and enhanced recovery in DSS-induced experimental colitis (Figures 7D,E and Figure S13 in Supplementary Material), and increased expression of M2 genes while decreasing expression of M1 genes (Figures 7F,G). These data, together with the indicated central role (Figure 8) of macrophages in miR-155-mediated protection of acute colitis, collectively support the therapeutic potential of anti-miR-155 based treatment in colitis.

## Discussion

Diverse regulatory mechanisms cooperate to maintain intestinal homeostasis, and breakdown in the intestinal epithelium or host immune system might lead to the pathology of colitis (40). Notably, many of these regulatory mechanisms are fine-tuned by the multifaceted regulator miR-155, which is expressed in a variety of immune cell types. We previously demonstrated that miR-155 in IECs acts as a negative regulator of intestinal innate tolerance during weaning transition (41). Besides, we also carefully monitored the colitis symptoms of miR-155^−/−^ mice throughout their life, under long-term SPF and dirty conditions, without observing spontaneous enteric inflammation (12).

Cre-mediated conditional gene deletion has been used to determine the contribution of genes in specific cell lineages (42). In our research, LysM-Cre mice were not used due to its known defect in the intestinal immune system (43). Gene deletion in LysM-Cre mice is targeted primarily to CD11b^+^ myeloid cells and not only macrophages but also including monocytes, neutrophils, and DCs. The adoptive transfer assays used instead demonstrated that miR-155 in macrophages, rather than in DCs, are primarily responsible for the induction and pathogenesis of acute colitis. This is probably due to the differential role of DCs and macrophages (17) in colon mucosal immunity or to the distinguished regulatory mechanisms and targets (44) of miR-155 in DCs and macrophages.

Our data demonstrated that the colon of miR-155^−/−^ mice displayed promoted M2 genes and reciprocally inhibited M1 genes in DSS colitis. This is consistent with the concept that plasticity is a fundamental characteristic of macrophages and that M1 and M2 are two extremes in a continuum of the polarization state (45). We speculated that a mixture of polarization phenotypes co-exists in the colon under M1-like conditions of DSS-induced colitis, and that miR-155 deficiency renders the dynamic equilibrium shift to M2 (Figure 8). Since our data show that miR-155 is required for circulating monocytes to differentiate into pro-inflammatory macrophages, we speculate that in acute colitis, monocytes recruited to the colon differentiate into inflammatory phenotypes under inflamed conditions. However, in the absence of miR-155, they might differentiate into cells with an anti-inflammatory M2-like phenotype, with function similar to tissue-resident macrophages (17). At the molecular level, we identified C/EBPβ and SOCS1 as two primary functional targets of miR-155 in macrophage polarization in inflamed conditions. Increased SOCS1 primarily contributes to the M1 program, whereas enhanced C/EBPβ is primarily responsible for the M2 program. Notably, there might be a positive link between miR-155 and IL-10 in macrophages, as IL-10 inhibits miR-155 induction (46), while miR-155 suppresses IL-10 production (47).

Although IL-10 and arginase activities were demonstrated as critical mediators of M2 in colitis, their cellular targets are unclear. We found that M2 might exert at least two critical actions in suppressing intestinal inflammation. First, M2 macrophages suppress the total number of immune cells in tissues via cell contact and/or the release of soluble mediators, as reported in acute lung injury (35). Second, M2 macrophages inhibit T cell Th1 and Th17 polarization through establishing a suppressive environment in colon.

The local immune environment is another determining factor in the dynamic equilibrium of macrophage functional plasticity (31). PEG2, rather than IL-4 or IL-13, was found in this study to play a critical role in potentiating the anti-inflammatory M2 phenotype in the early stage of colitis in miR-155^−/−^ mice. Indeed, PEG2 phosphorylation (48) of CREB leads to increased transcription of C/EBPβ, a primary functional target of miR-155 in M2 polarization.

In spite of PGE2 contributing to the initiation of M2 macrophage polarization, we speculate that Th2 cytokines, such as IL-4, may further facilitate M2 polarization in the late stage of colitis in miR-155^−/−^ mice, as miR-155^−/−^ CD4+ T cells were intrinsically more prone to polarize toward Th2 cells with amplified Th2 cytokine production (12). From the perspective of evolution, the miR-155 gene may have evolved as protection against infectious disease, fitting with its related upregulation in modern-day organisms. Accordingly, just as a double-edged sword, miR-155 can be harmful in inflammatory diseases, such as colitis. In conclusion, our study, which used a rational approach based on anti-mir-155, suggests that specific targeting of macrophages may achieve ideal therapeutic effects in intestinal inflammation.

## Ethics Statement

All mouse work was done according to the requirements of Third Military Medical University Animal Ethics Committee (Approval number TMMU 08-08-01). Animals were sacrificed using CO_2_ asphyxiation and the appropriate organs harvested. All of the patients provided written informed consent to participate in the study, and all related procedures were carried out in accordance with the principles of the Declaration of Helsinki; the human aspect of the study was approved by the ethics committee of Xin Qiao Hospital, AMU.

## Author Contributions

JL, JZ, and YW designed and discussed the study. HG and HoL carried out most of the experiments and collected and analyzed the data. GA and HH performed the mice experiments. SY and PY performed the experiments involving clinical samples. WF and NY performed the cytokine detection. TY contributed to WB and Q-PCR experiments. ZT, ZS, HuL, and XL contributed to the cell experiments. JL and JZ contributed to the writing of the paper.

## Conflict of Interest Statement

The authors declare that the research was conducted in the absence of any commercial or financial relationships that could be construed as a potential conflict of interest.

## Supplementary Material

Figure S1 Expression level of miR-155 is increased in colon tissue of UC patients and mice treated with dextran sulfate sodium (DSS). WT (*n* = 10) mice were given 3% DSS in drinking water for 5 days, followed by regular drinking water for 6 days. **(A–C)** RNA was isolated from colon tissues **(A)**, isolated IECs **(B)**, and LPMCs **(C)** of mice, and miR-155 expression was analyzed by Q-PCR. **(D)** RNA was isolated from fresh colon tissues of HC patients (*n* = 20), inflamed mucosa of patients with active UC (*n* = 22) patients and miR-155 expression was analyzed by Q-PCR. **P* < 0.05, ***P* < 0.01 vs water control (Student’s *t*-test). Data are representative of two independent experiments [mean and SD in **(A–D)**]. HC, healthy control; UC, ulcerative colitis; WT, wild-type.

Figure S2 miR-155^−/−^ mice showed attenuated disease severity in TNBS-mediated colitis. WT mice (*n* = 12) and miR-155^−/−^ (*n* = 13) mice and were subjected to rectal injection of TNBS and body weight **(A)** and survival **(B)** were monitored. **P* < 0.05, ***P* < 0.01 vs WT control [Student’s *t*-test in a and Kaplan–Meier analysis in **(B)**]. Data are representative of two independent experiments (mean and SD). WT, wild-type; TNBS, 2,4,6-trinitrobenzene sulfonic acid.

Figure S3 Analysis of microbiota composition in WT and miR-155^−/−^ mice. Stool genomic DNA of 8-week-old WT and miR-155^−/−^ mice was extracted and numbers of total bacteria, *Enterobacteriaceae* bacteria (Ent), the *E. rectale–C. coccoides* (Eub) group bacteria, and the *Bacteroides* (Bac) group bacteria per gram of stool were quantified by Q-PCR. ns vs WT control. Data are representative of three independent experiments [mean and SEM in **(A–D)**]; *n* = 12–15 mice per group. ns, not significant; WT, wild-type.

Figure S4 No significant difference in epithelial permeability, epithelial cell proliferation or apoptosis between WT and miR-155^−/−^ mice. WT (*n* = 10) or miR-155^−/−^ (*n* = 10) mice were given 3% dextran sulfate sodium (DSS) in drinking water for 5 days, followed by regular drinking water for 6 days (DSS condition) or regular drinking water for 11 days (water condition). In both conditions, **(A)** WT or miR-155^−/−^ mice were fed with FITC-dextran, and FITC-dextran amounts in serum were determined 3 h later. **(B)** WT or miR-155^−/−^ mice were injected intraperitoneally with BrdU, and the number of BrdU-positive cells was counted. **(C)** *In situ* TUNEL assay was performed on colon sections of WT or miR-155^−/−^ mice, and apoptotic cells/5 crypts were counted. ns vs WT control (Student’s *t*-test). Data are representative of two independent experiments (mean and SD). ns, not significant. WT, wild-type.

Figure S5 miR-155^−/−^ mice showed reduced pro-inflammatory cytokines and inflammatory cell infiltration in colon following dextran sulfate sodium (DSS) challenge. WT (*n* = 3) or miR-155^−/−^ (*n* = 3) mice were given 3% DSS in drinking water for 5 days, followed by regular drinking water for 6 days. **(A)** Total RNA was extracted from colon tissues and were analyzed by the Innate & Adaptive Immune Responses PCR Array kit (Qiagen); the pro-inflammatory genes (left); and anti-inflammatory genes (right) among the differentially expressed genes are shown, respectively. **(B)** Colon tissue was cultured for 24 h, and cytokines in supernatants were analyzed by ELISA and then normalized by total colon tissue weight of whole-colon culture. **P* < 0.05, ***P* < 0.01 vs WT control (Student’s *t*-test). Data are representative of two independent experiments (mean and SD). LPMCs, lamina propria mononuclear cells; WT, wild-type.

Figure S6 Confirmation of bone marrow chimeric mice reconstitution. PBMCs of the chimeric mice stained with anti-CD45.1-APC (for WT mice) and anti-CD45.2-FITC (for miR-155^−/−^ mice) detected by FACS. WT, wild-type.

Figure S7 The representatives picture of colon length of bone marrow chimeras mice.

Figure S8 Confirmation of gut macrophages deletion by clodronate-liposomes. The LPMCs were isolated from colon tissues of PBS-liposomes or clodronate-liposomes treated mice were analyzed by flow cytometry. The representative FACS shows the frequency of F4/80^+^ positive macrophages from both WT and miR-155^−/−^ mice.

Figure S9 Gating strategy of circulating Ly6Chi monocytes (CD45^+^CD115^+^Ly6G-Ly6C^hi^) sorting from mice blood.

Figure S10 Flow cytometry-sorted mouse blood monocytes (CD45^+^CD115^+^Ly6G-Ly6C^hi^) were stimulated in M1 mimic condition (10 µg/mL CBA and 20 ng/mL IFN-γ, * M1 condition) and M2 condition (15 ng/mL IL-4, * M2 condition) *in vitro*. In both conditions, the relative expressions of mRNA associated with M1-polarized macrophages and M2-polarized macrophages were measured by Q-PCR. **P* < 0.05, ***P* < 0.01 vs WT control (Student’s *t*-test).

Figure S11 miR-155^−/−^ mice (*n* = 5/group) were i.p. injected with anti-IL-4 and/or anti-IL-13 neutralizing antibody at dextran sulfate sodium (DSS) treatment days 1, 3, and 5, and the relative expression of M2 genes were measured by Q-PCR. ns as determined by ANOVA with Bonferroni’s posttest correction for multiple comparisons. Data are representative of two or three independent experiments (mean and SD). ns, not significant; WT, wild-type.

Figure S12 Macrophages were transfected with C/EBP β siRNA, SOCS-1 siRNA, SHIP-1 siRNA and their control, respectively, and cultured for 48 h, the mRNA expression of C/EBPβ **(A)**, SOCS-1 **(B)**, and SHIP-1 **(C)** were determined by Q-PCR, and the protein expression was analyzed by Western blot.

Figure S13 The body weight of WT mice with dextran sulfate sodium (DSS) colitis that were treated with antagomir-155 or of NC mice were monitored daily till day 15 after DSS challenge. **P* < 0.05, vs antagomir-treated NC (Student’s *t*-test); *n* = 4–5 per group. Data are representative of two independent experiments (mean and SD). NC, negative control; WT, wild-type.
