# Corrigendum: Critical Role of Alternative M2 Skewing in miR-155 Deletion-Mediated Protection of Colitis

**DOI:** 10.3389/fimmu.2019.03153

**Published:** 2020-02-13

**Authors:** Jintao Li, Ji Zhang, Hongxia Guo, Shimin Yang, Weiping Fan, Nan Ye, Zhiqiang Tian, Tiantian Yu, Guoping Ai, Zigang Shen, Haiyang He, Ping Yan, Hui Lin, Xue Luo, Hongli Li, Yuzhang Wu

**Affiliations:** ^1^Institute of Tropical Medicine, Army Medical University, Chongqing, China; ^2^Department of Microbiology, College of Basic Medicine, Army Medical University, Chongqing, China; ^3^Institute of Immunology, PLA, Army Medical University, Chongqing, China; ^4^Department of Gastroenterology, Xinqiao Hospital, Army Medical University, Chongqing, China; ^5^Department of Microbiology and Immunology, Shanxi Medical University, Taiyuan, China; ^6^Department of Obstetrics and Gynecology, Southwest Hospital, Army Medical University, Chongqing, China; ^7^Department of Histology and Embryology, College of Basic Medicine, Army Medical University, Chongqing, China

**Keywords:** M2 macrophages, miR-155, colitis, C/EBPβ, SOCS1

In the original article, there were mistakes in Figure 1J and Figure 6E as published. Figure 1J (representative FACS plot of CD4^+^IFN-γ^+^ cells) was mistakenly duplicated from Figure 1I (representative FACS plot of CD4^+^IL-17^+^ cells), and the tubulin band of Figure 6E was inadvertently covered by the band of that in Figure 6C. The corrected Figure 1 and Figure 6 appear below.

**Figure 1:**
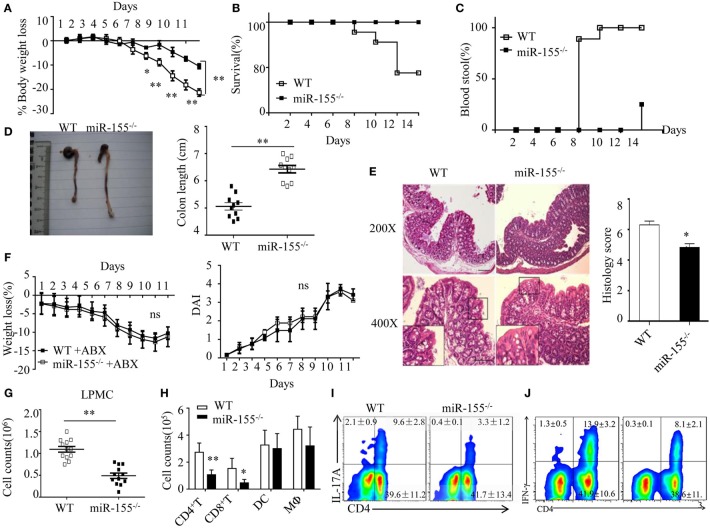
Attenuated dextran sulfate sodium (DSS)-induced colitis in miR-155^−/−^ mice is dependent on commensal bacteria. WT or miR-155^−/−^ mice were given 3% DSS in drinking water for 5 days, followed by regular drinking water for 6 days. **(A–E)** Weight change **(A)**, Kaplan–Meier plot of survival rate **(B)**, stool blood **(C)**, representative gross colon appearance [**(D)** left] and colon length [**(D)** right], and representative H&E-stained colon cross-sections [**(E)** left, original magnification, 200× or 400×] and semi-quantitative histopathology score [**(E)** right]. **(F)** WT and miR-155^−/−^ mice were treated with broad-spectrum antibiotics cocktail (ABX) for 4 weeks and then given 3% DSS, the body weight change (left) and DAI (right) were monitored daily. Ns vs WT control **(G,H)**. The LPMCs were isolated from colon tissues of DSS-treated WT (*n* = 12) and miR-155^−/−^ (*n* = 15) mice, then the total number of LPMCs (CD45^+^) **(G)**, T cells (CD4^+^ and CD8^+^), DCs (CD11c^+^CD11b^−^) and macrophages (CD11b^+^CD11c^−/low^) **(H)** were counted by flow cytometry. **(I,J)** Representative FACS showing CD4^+^IL-17^+^ cells **(I)** and CD4^+^IFN-γ^+^ cells **(J)** in isolated LPMCs of DSS-treated WT (*n* = 12) and miR-155^−/−^ (*n* = 15) mice. *P < 0.05, **P < 0.01 vs WT control [Student's *t*-test in **(A,D,E,G,H)** and Kaplan–Meier analysis in **(B,C)**]. ns vs WT control. Data are representative of three independent experiments (mean and SD in A-D); *n* = 12–15 mice per group in **(A–F)** and *n* = 5–6 mice per group in **(G)**. ns, not significant. WT, wild-type.

**Figure 6:**
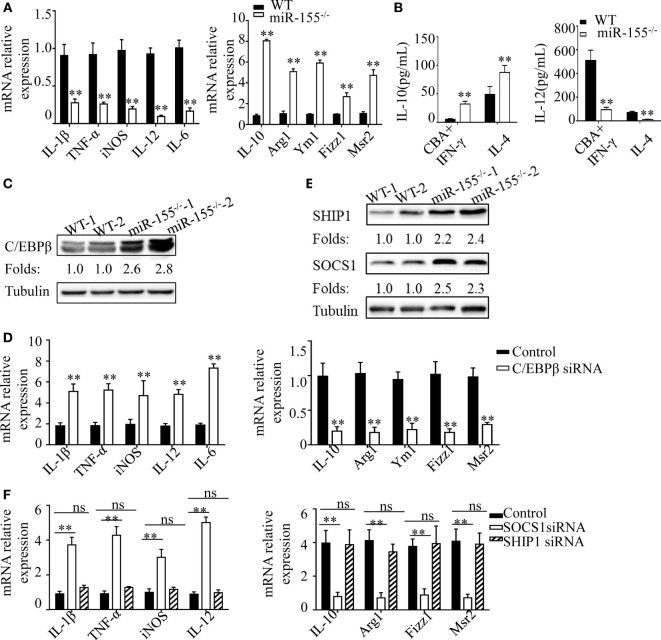
C/EBPβ and SOCS1 are key functional targets in intestinal M2 polarization. **(A)** BMDMs isolated from WT and miR-155^−/−^ mice were treated with CBA (10 μg/mL) and IFN-γ (20 ng/mL), and the relative expression of M1genes and M2 genes were determined by Q-PCR. **(B)** The absolute amounts of secreted cytokines IL-10 and IL-12 (as representative of M2 and M1 gene products, respectively) in the supernatants of WT or miR-155^−/−^ BMDMs that had been treated with M1 condition (CBA + IFN-γ) and M2 condition (IL-4) were measured by ELISA. **(C)** The protein expression level of C/EBPβ in macrophages (CD11b^+^CD11c^−/low^) isolated from LPMCs of dextran sulfate sodium colitis mice were determined by western blotting. **(D)** miR-155^−/−^ BMDMs were transferred with C/EBPβ siRNA or control and then stimulated with CBA (10 μg/mL) and IFN-γ (20 ng/mL), and the relative expression of M1genes and M2 genes were determined by Q-PCR. **(E)** The protein expression level of SOCS1 and SHIP1 in macrophages, as described in **(C)**, was determined by western blotting. **(F)** miR-155^−/−^ BMDMs were transferred with SOCS1 and SHIP1 siRNA and treated as described in **(D)**, and the relative expressions of M1genes and M2 genes were determined by Q-PCR. *P < 0.05, **P < 0.01 vs WT control or siRNA control [Student's *t*-test in **(A,B,D)**]. *P < 0.05, ns > 0.05 vs. siRNA control (ANOVA with Bonferroni's posttest correction for multiple comparisons in **(F)**. Data are representative of three independent experiments (mean and SD). Ns, not significant. BMDMs, bone marrow-derived macrophage. WT, wild-type. CBA, cecal bacterial antigen.

The authors apologize for this error and state that this does not change the scientific conclusions of the article in any way. The original article has been updated.

