# Supplementary figures and images for: Critical Role of Alternative M2 Skewing in miR-155 Deletion-Mediated Protection of Colitis
Source: Front Immunol. 2018 May 3;9:904. doi: 10.3389/fimmu.2018.00904 (PMC5943557; doi:10.3389/fimmu.2018.00904)

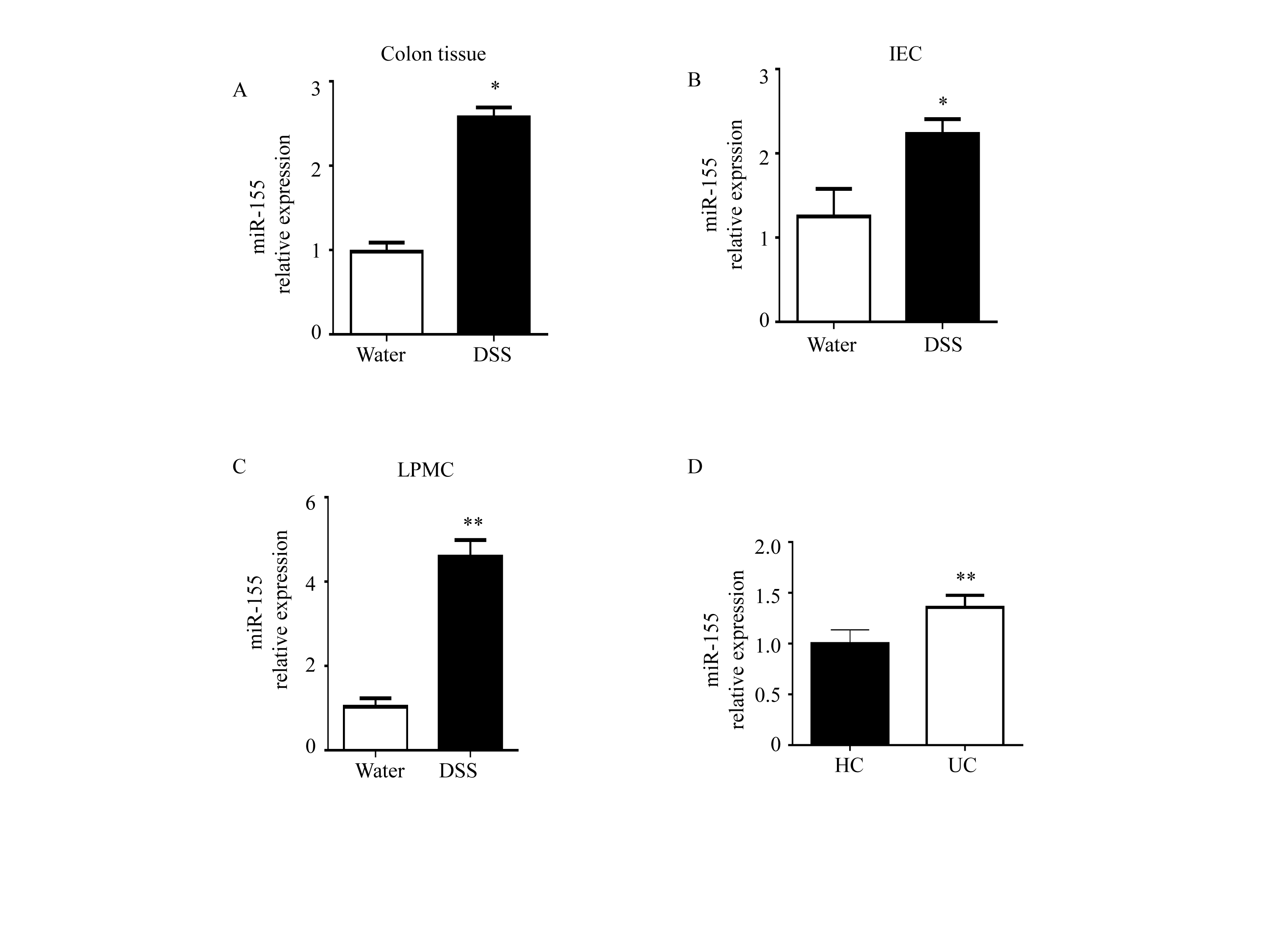

Supplement: Figure S1 — Expression level of miR-155 is increased in colon tissue of UC patients and mice treated with dextran sulfate sodium (DSS). WT (n = 10) mice were given 3% DSS in drinking water for 5 days, followed by regular drinking water for 6 days. (A–C) RNA was isolated from colon tissues (A), isolated IECs (B), and LPMCs (C) of mice, and miR-155 expression was analyzed by Q-PCR. (D) RNA was isolated from fresh colon tissues of HC patients (n = 20), inflamed mucosa of patients with active UC (n = 22) patients and miR-155 expression was analyzed by Q-PCR. *P < 0.05, **P < 0.01 vs water control (Student’s t-test). Data are representative of two independent experiments [mean and SD in (A–D)]. HC, healthy control; UC, ulcerative colitis; WT, wild-type. [file Image_1.tif]

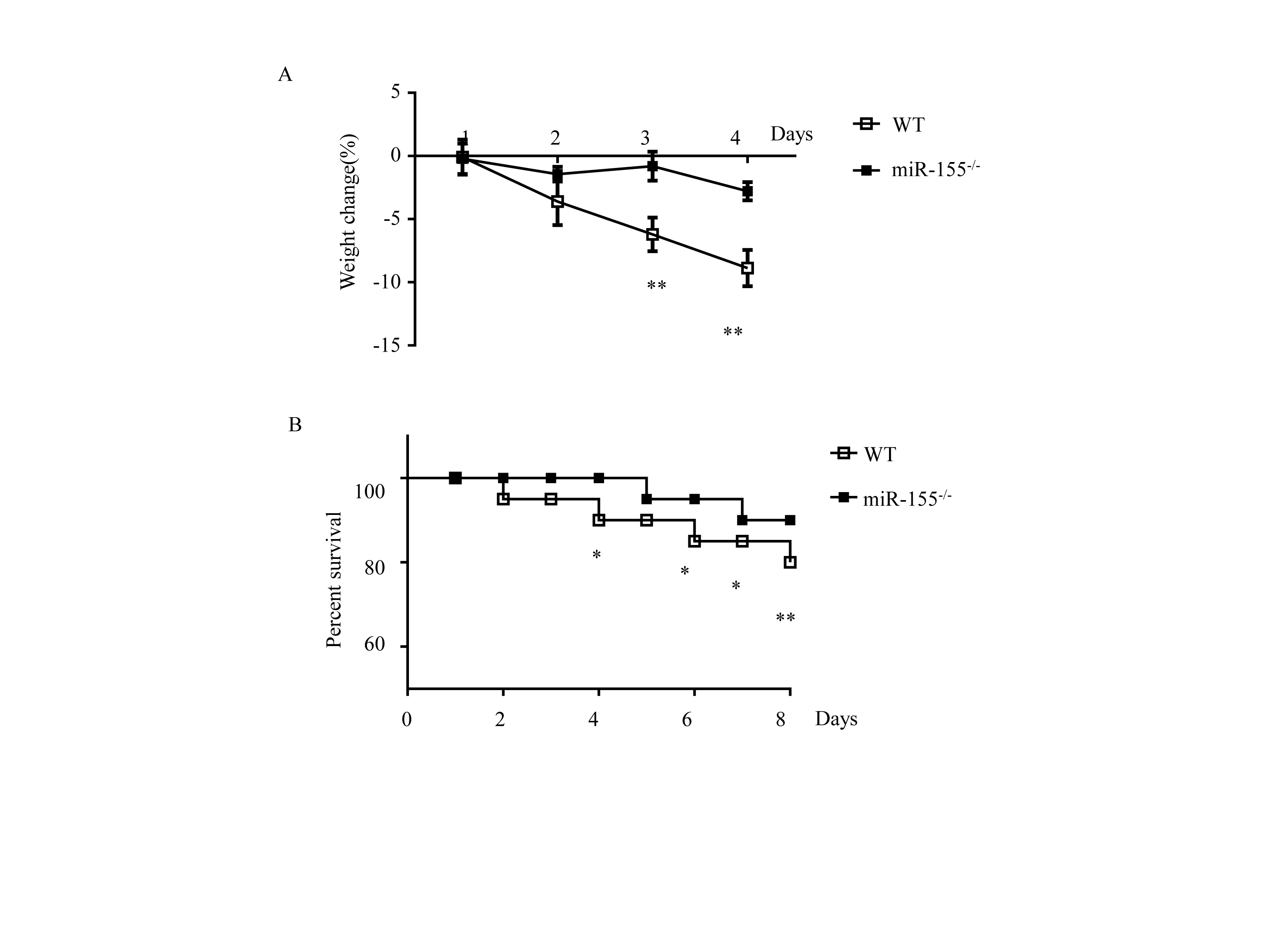

Supplement: Figure S2 — miR-155−/− mice showed attenuated disease severity in TNBS-mediated colitis. WT mice (n = 12) and miR-155−/− (n = 13) mice and were subjected to rectal injection of TNBS and body weight (A) and survival (B) were monitored. *P < 0.05, **P < 0.01 vs WT control [Student’s t-test in a and Kaplan–Meier analysis in (B)]. Data are representative of two independent experiments (mean and SD). WT, wild-type; TNBS, 2,4,6-trinitrobenzene sulfonic acid. [file Image_2.tif]

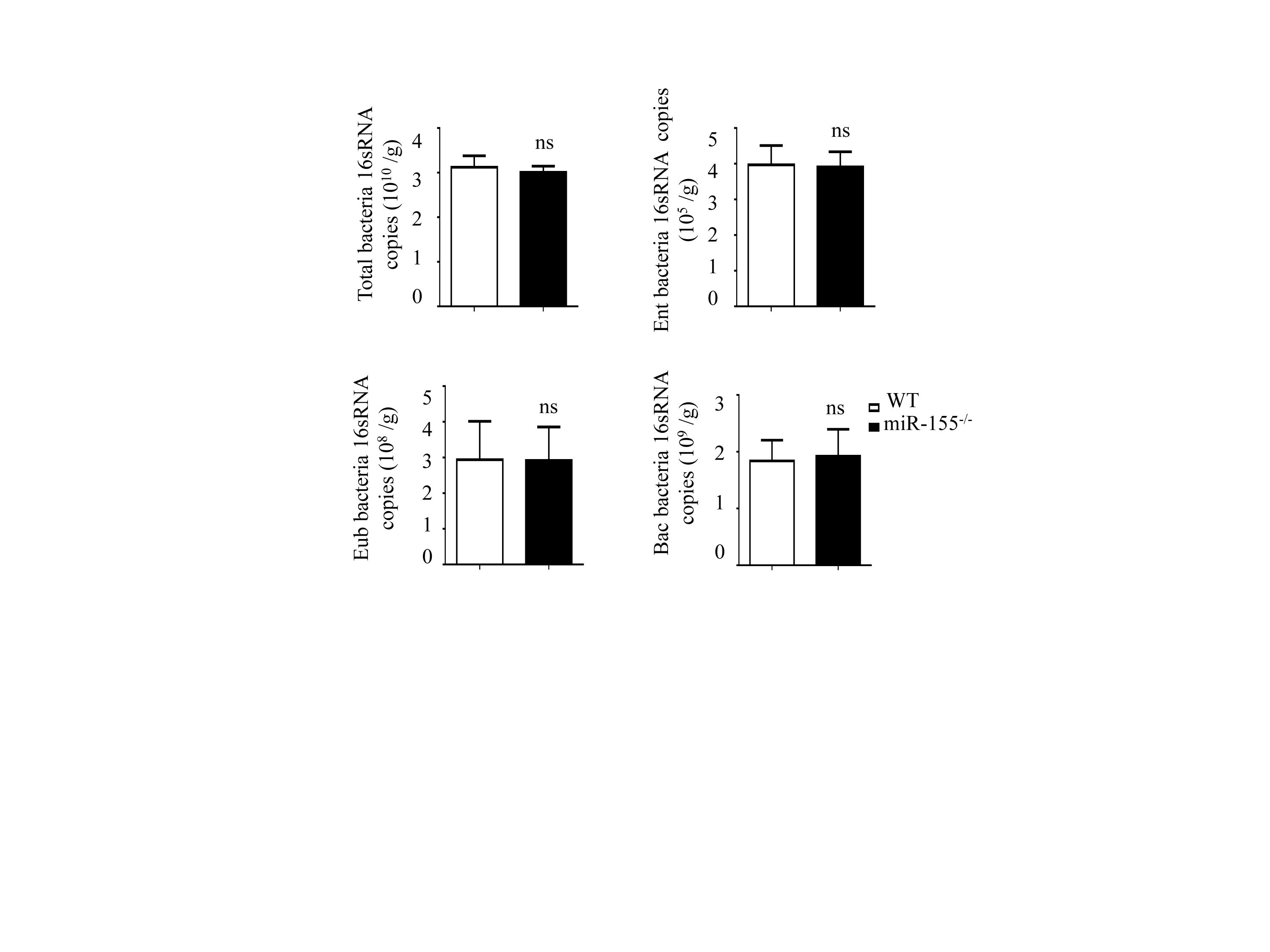

Supplement: Figure S3 — Analysis of microbiota composition in WT and miR-155−/− mice. Stool genomic DNA of 8-week-old WT and miR-155−/− mice was extracted and numbers of total bacteria, Enterobacteriaceae bacteria (Ent), the E. rectale–C. coccoides (Eub) group bacteria, and the Bacteroides (Bac) group bacteria per gram of stool were quantified by Q-PCR. ns vs WT control. Data are representative of three independent experiments [mean and SEM in (A–D)]; n = 12–15 mice per group. ns, not significant; WT, wild-type. [file Image_3.tif]

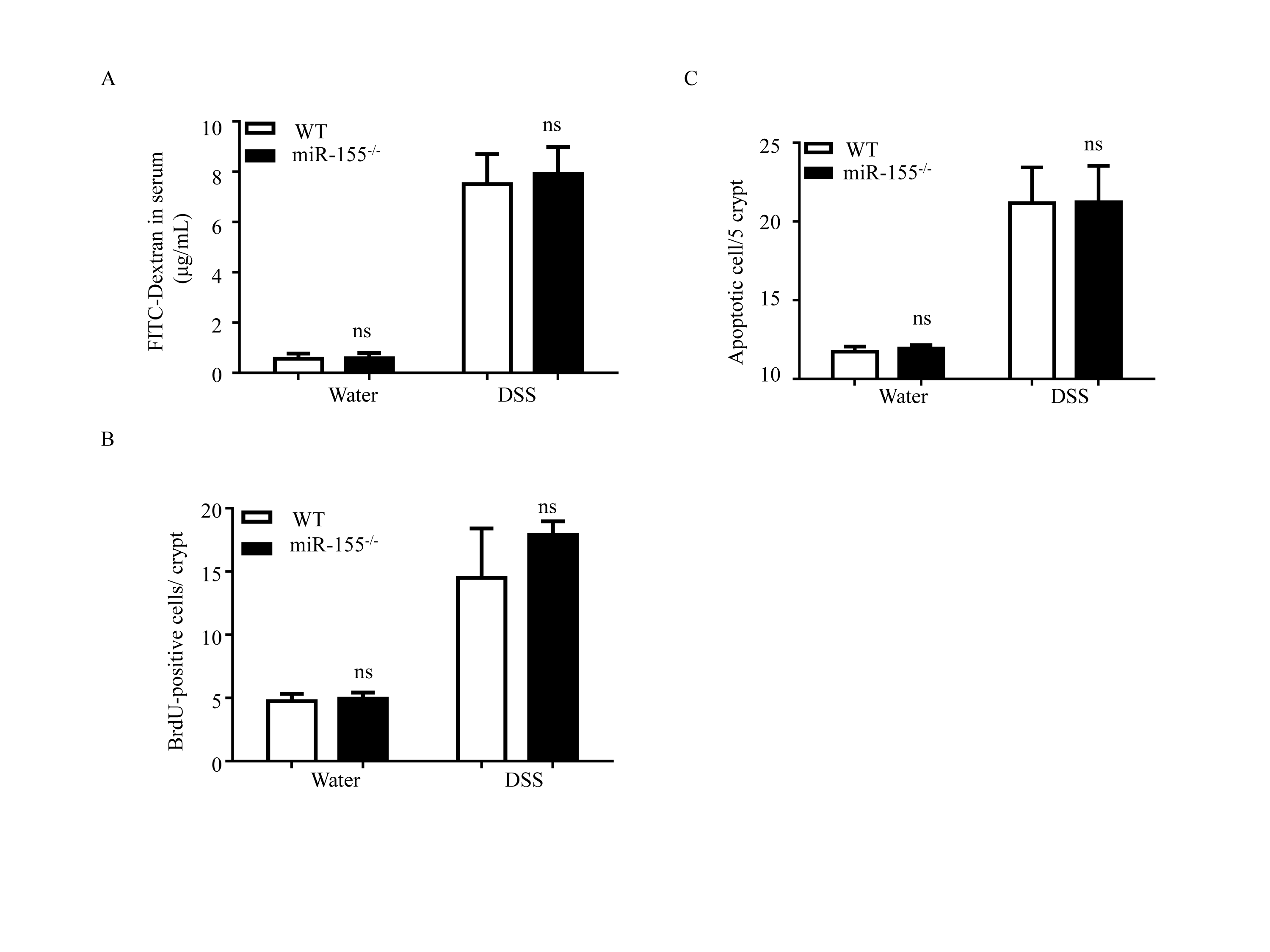

Supplement: Figure S4 — No significant difference in epithelial permeability, epithelial cell proliferation or apoptosis between WT and miR-155−/− mice. WT (n = 10) or miR-155−/− (n = 10) mice were given 3% dextran sulfate sodium (DSS) in drinking water for 5 days, followed by regular drinking water for 6 days (DSS condition) or regular drinking water for 11 days (water condition). In both conditions, (A) WT or miR-155−/− mice were fed with FITC-dextran, and FITC-dextran amounts in serum were determined 3 h later. (B) WT or miR-155−/− mice were injected intraperitoneally with BrdU, and the number of BrdU-positive cells was counted. (C) In situ TUNEL assay was performed on colon sections of WT or miR-155−/− mice, and apoptotic cells/5 crypts were counted. ns vs WT control (Student’s t-test). Data are representative of two independent experiments (mean and SD). ns, not significant. WT, wild-type. [file Image_4.tif]

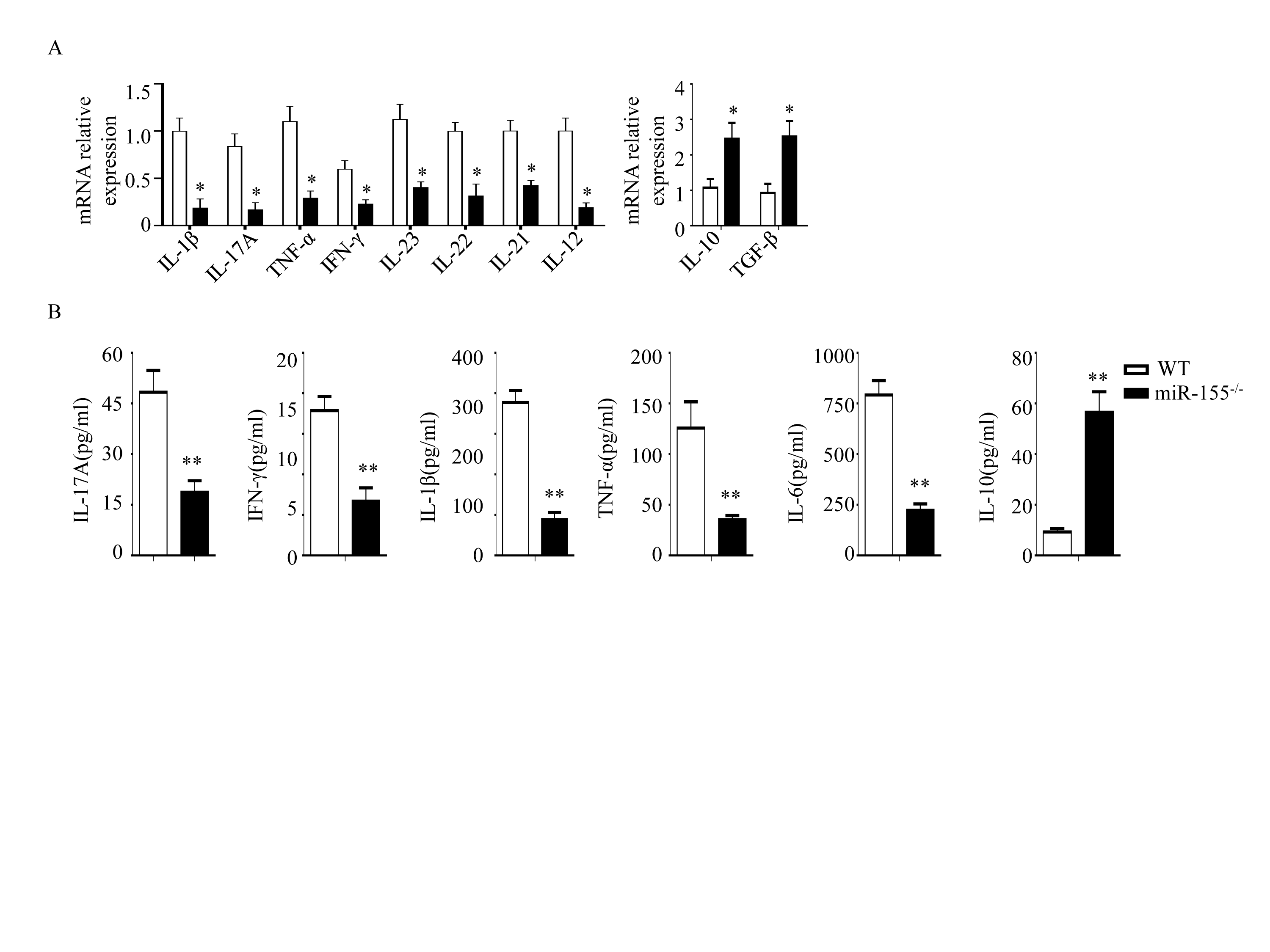

Supplement: Figure S5 — miR-155−/− mice showed reduced pro-inflammatory cytokines and inflammatory cell infiltration in colon following dextran sulfate sodium (DSS) challenge. WT (n = 3) or miR-155−/− (n = 3) mice were given 3% DSS in drinking water for 5 days, followed by regular drinking water for 6 days. (A) Total RNA was extracted from colon tissues and were analyzed by the Innate & Adaptive Immune Responses PCR Array kit (Qiagen); the pro-inflammatory genes (left); and anti-inflammatory genes (right) among the differentially expressed genes are shown, respectively. (B) Colon tissue was cultured for 24 h, and cytokines in supernatants were analyzed by ELISA and then normalized by total colon tissue weight of whole-colon culture. *P < 0.05, **P < 0.01 vs WT control (Student’s t-test). Data are representative of two independent experiments (mean and SD). LPMCs, lamina propria mononuclear cells; WT, wild-type. [file Image_5.tif]

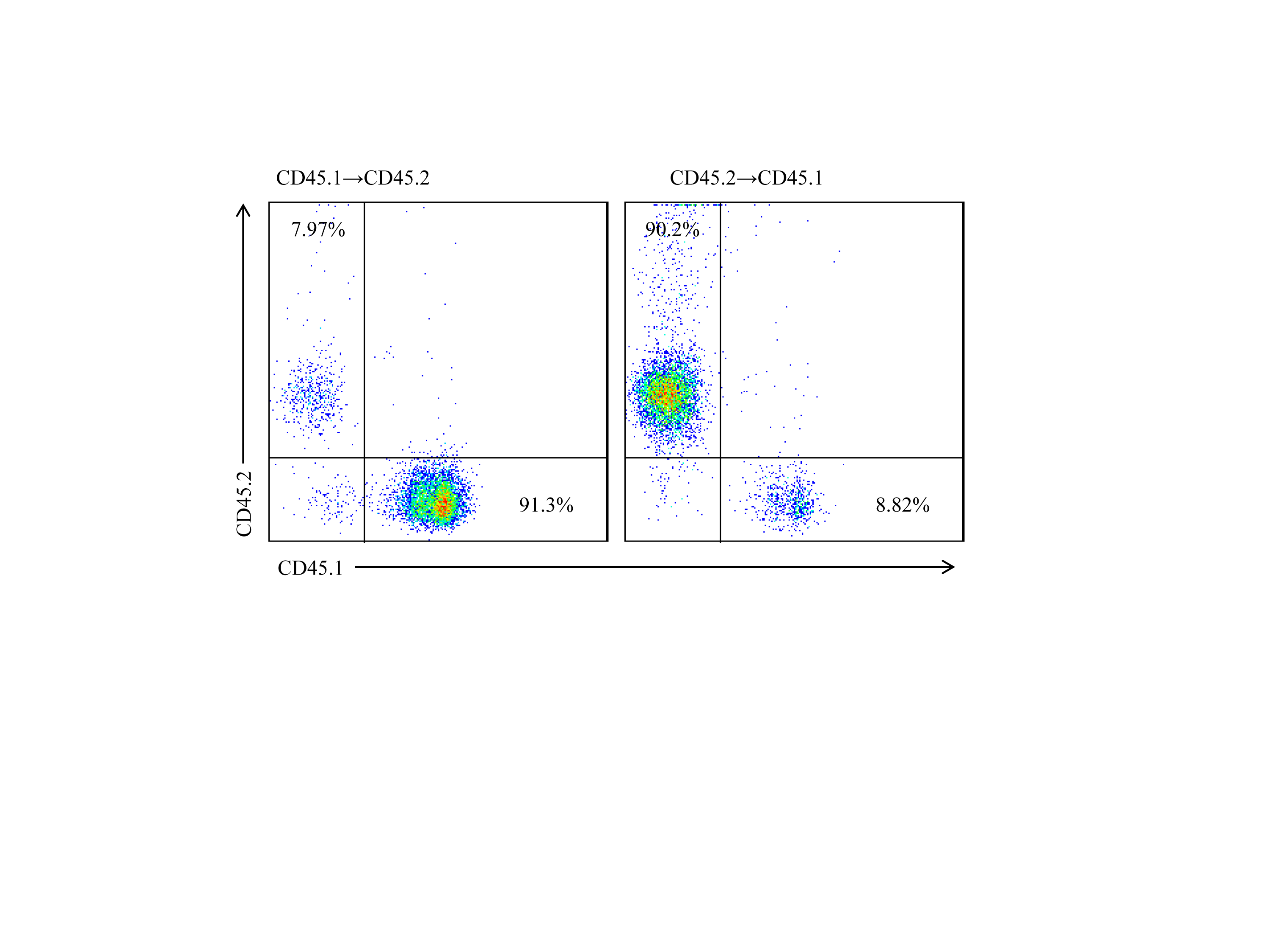

Supplement: Figure S6 — Confirmation of bone marrow chimeric mice reconstitution. PBMCs of the chimeric mice stained with anti-CD45.1-APC (for WT mice) and anti-CD45.2-FITC (for miR-155−/− mice) detected by FACS. WT, wild-type. [file Image_6.tif]

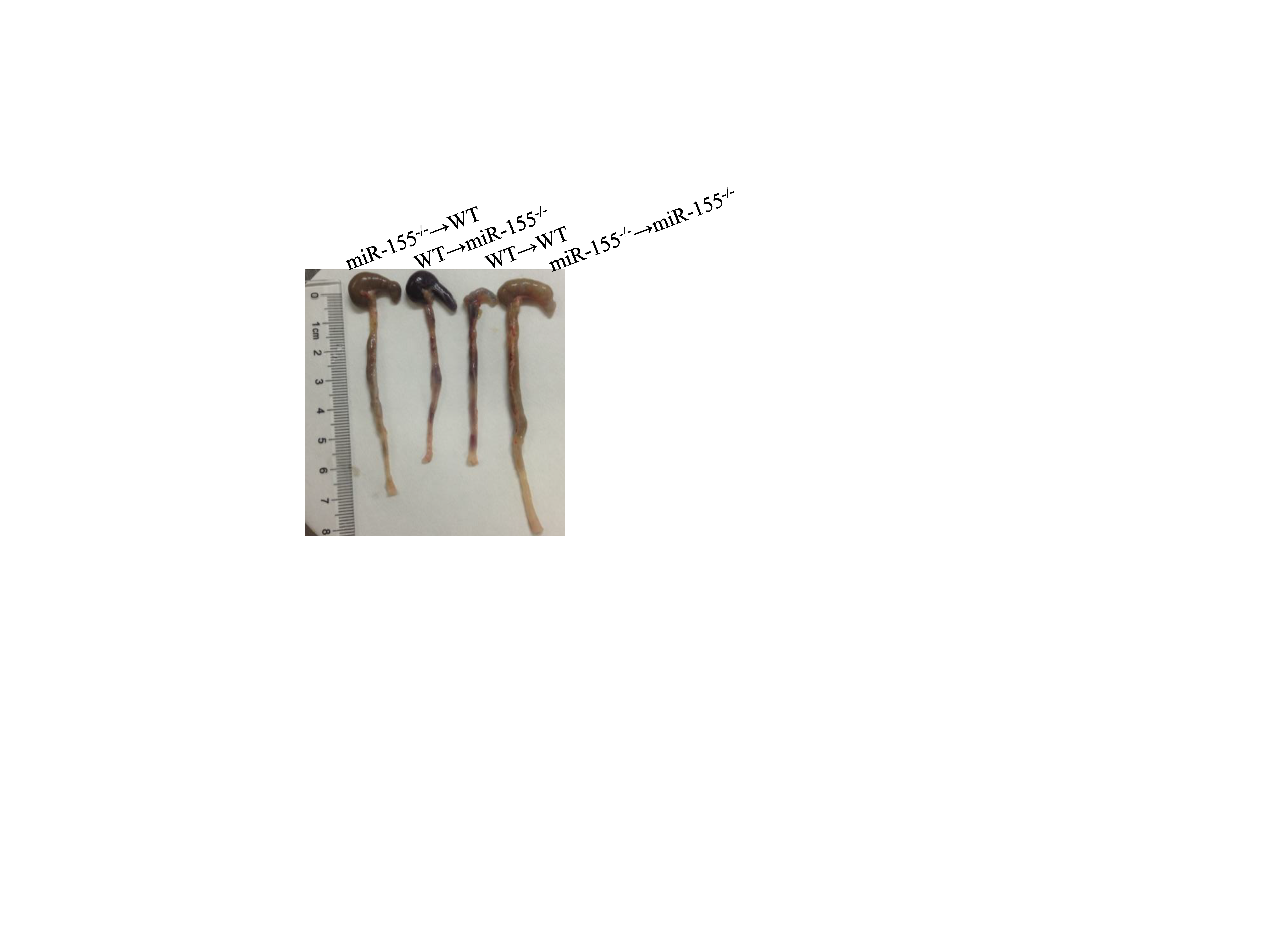

Supplement: Figure S7 — The representatives picture of colon length of bone marrow chimeras mice. [file Image_7.tiff]

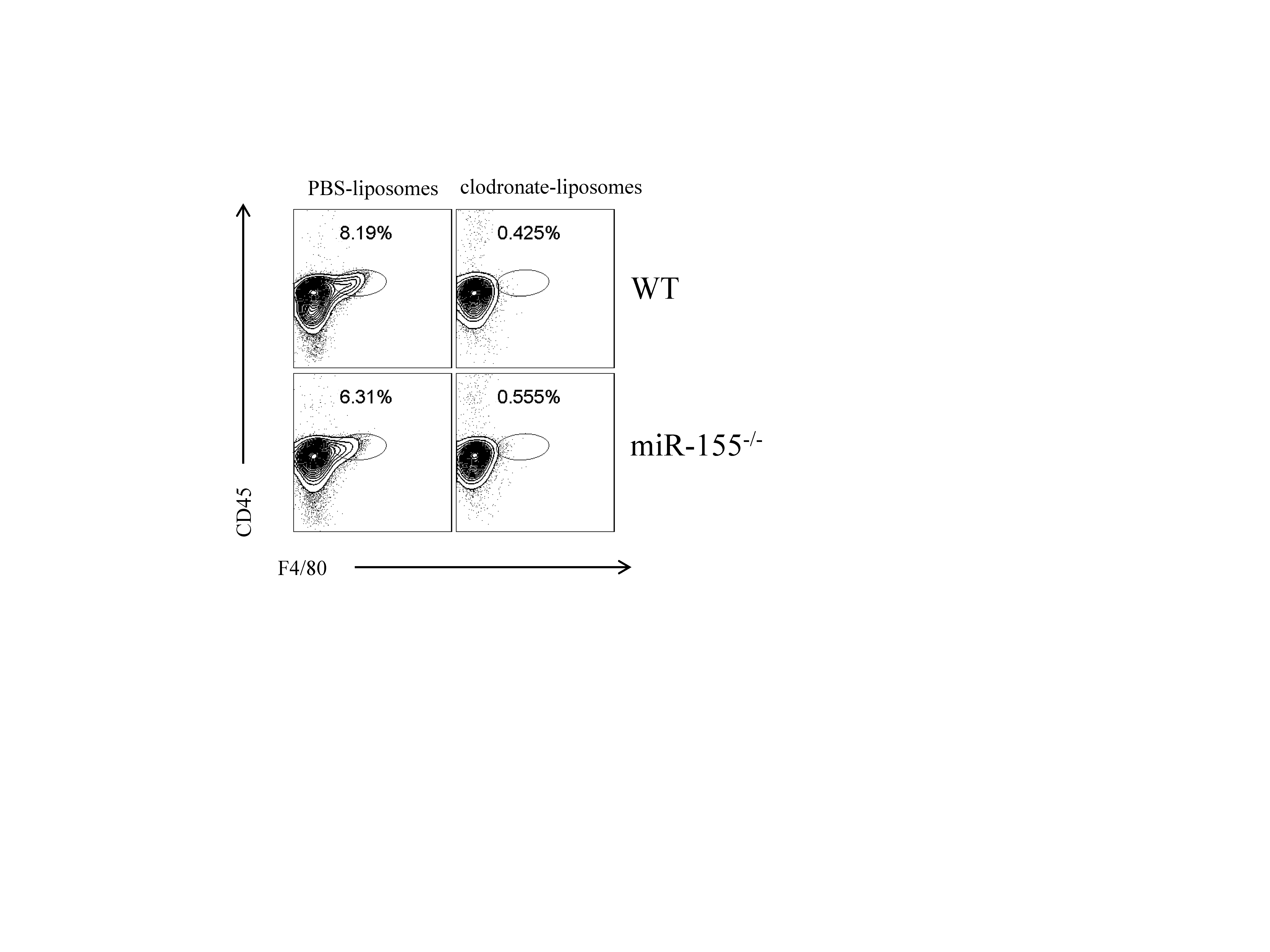

Supplement: Figure S8 — Confirmation of gut macrophages deletion by clodronate-liposomes. The LPMCs were isolated from colon tissues of PBS-liposomes or clodronate-liposomes treated mice were analyzed by flow cytometry. The representative FACS shows the frequency of F4/80+ positive macrophages from both WT and miR-155−/− mice. [file Image_8.tiff]

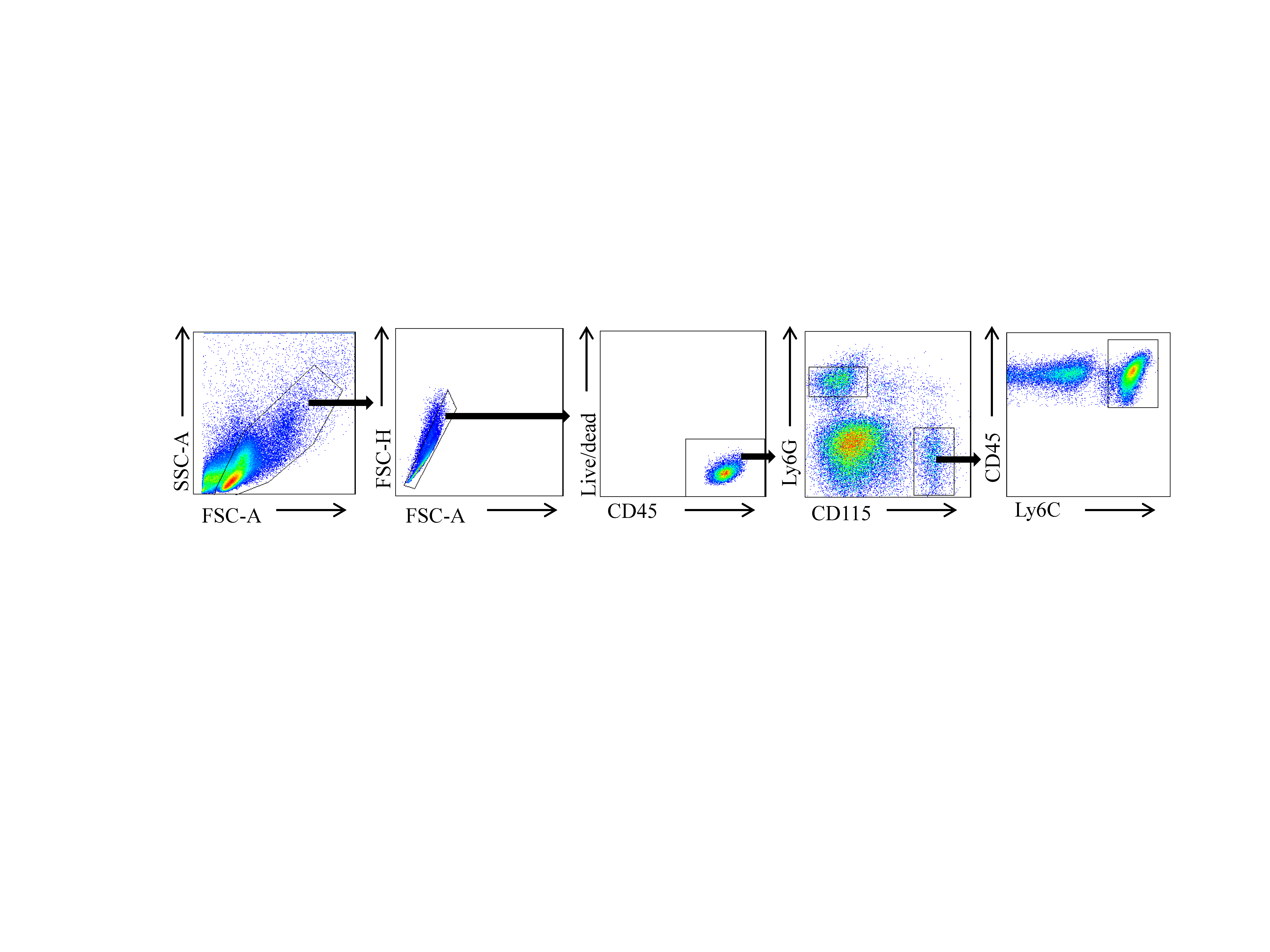

Supplement: Figure S9 — Gating strategy of circulating Ly6Chi monocytes (CD45+CD115+Ly6G-Ly6Chi) sorting from mice blood. [file Image_9.tiff]

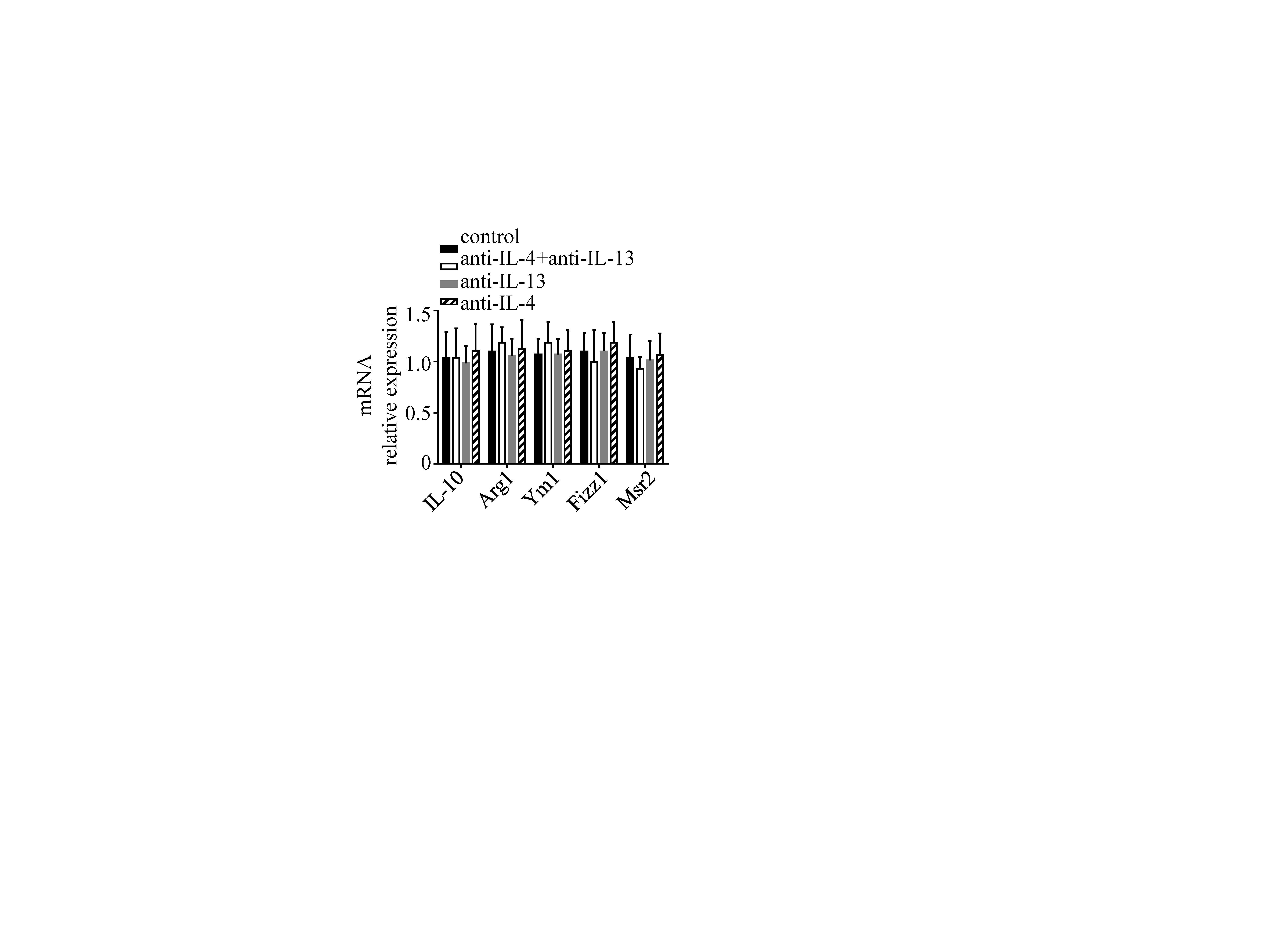

Supplement: Figure S11 — miR-155−/− mice (n = 5/group) were i.p. injected with anti-IL-4 and/or anti-IL-13 neutralizing antibody at dextran sulfate sodium (DSS) treatment days 1, 3, and 5, and the relative expression of M2 genes were measured by Q-PCR. ns as determined by ANOVA with Bonferroni’s posttest correction for multiple comparisons. Data are representative of two or three independent experiments (mean and SD). ns, not significant; WT, wild-type. [file Image_11.tiff]

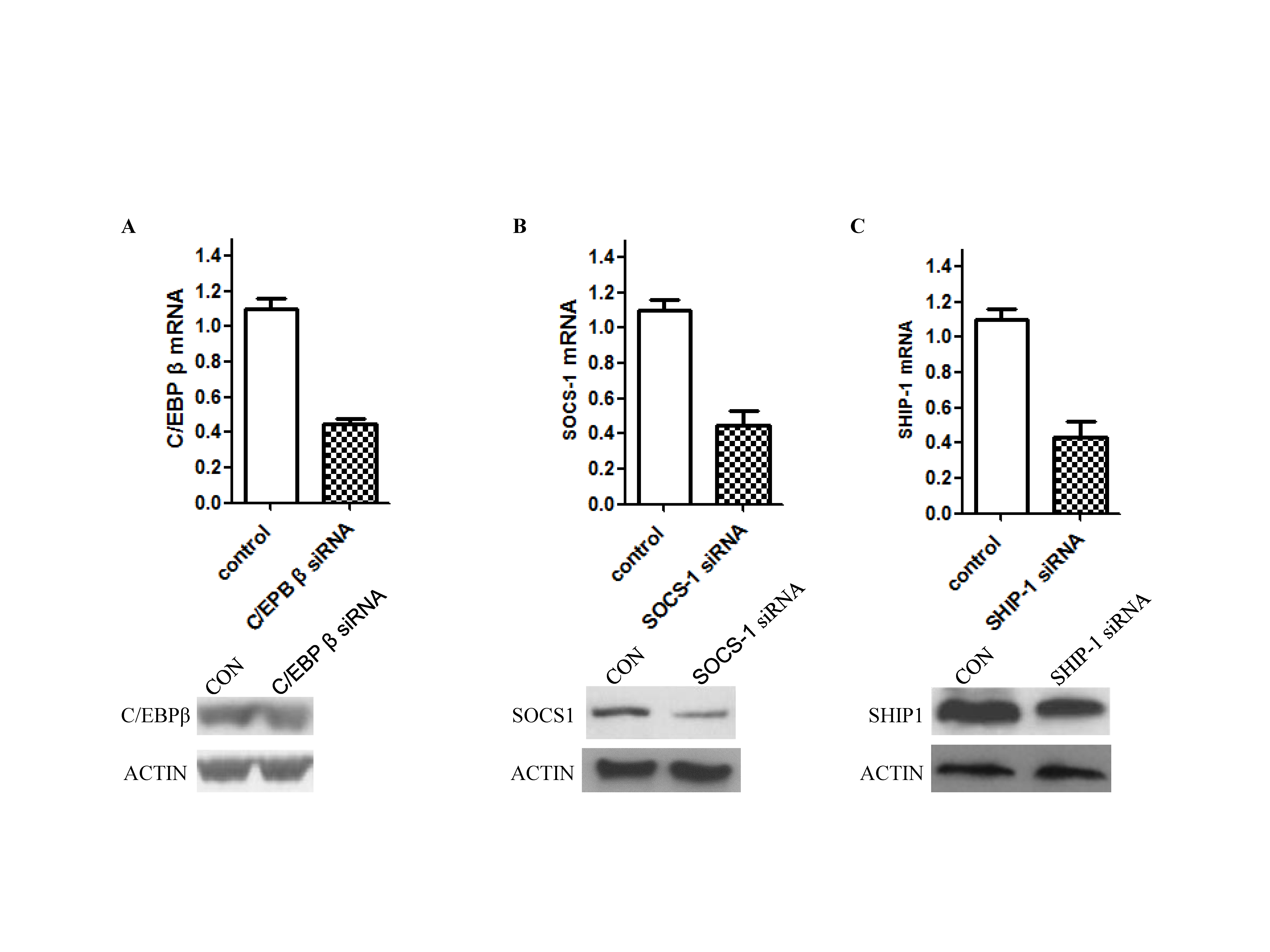

Supplement: Figure S12 — Macrophages were transfected with C/EBP β siRNA, SOCS-1 siRNA, SHIP-1 siRNA and their control, respectively, and cultured for 48 h, the mRNA expression of C/EBPβ (A), SOCS-1 (B), and SHIP-1 (C) were determined by Q-PCR, and the protein expression was analyzed by Western blot. [file Image_12.tiff]
